# Identification of New Genes Contributing to the Extreme Radioresistance of *Deinococcus radiodurans* Using a Tn5-Based Transposon Mutant Library

**DOI:** 10.1371/journal.pone.0124358

**Published:** 2015-04-17

**Authors:** Rémi Dulermo, Takefumi Onodera, Geneviève Coste, Fanny Passot, Murielle Dutertre, Martine Porteron, Fabrice Confalonieri, Suzanne Sommer, Cécile Pasternak

**Affiliations:** Univ. Paris-Sud, Institute for Integrative Biology of the Cell (I2BC), Université Paris Saclay, CEA, CNRS, Orsay, France; Louisiana State University and A & M College, UNITED STATES

## Abstract

Here, we have developed an extremely efficient *in vivo* Tn5-based mutagenesis procedure to construct a *Deinococcus radiodurans* insertion mutant library subsequently screened for sensitivity to genotoxic agents such as γ and UV radiations or mitomycin C. The genes inactivated in radiosensitive mutants belong to various functional categories, including DNA repair functions, stress responses, signal transduction, membrane transport, several metabolic pathways, and genes of unknown function. Interestingly, preliminary characterization of previously undescribed radiosensitive mutants suggests the contribution of cyclic di-AMP signaling in the recovery of *D*. *radiodurans* cells from genotoxic stresses, probably by modulating several pathways involved in the overall cell response. Our analyses also point out a new transcriptional regulator belonging to the GntR family, encoded by *DR0265*, and a predicted RNase belonging to the newly described Y family, both contributing to the extreme radioresistance of *D*. *radiodurans*. Altogether, this work has revealed new cell responses involved either directly or indirectly in repair of various cell damage and confirmed that *D*. *radiodurans* extreme radiation resistance is determined by a multiplicity of pathways acting as a complex network.

## Introduction

The extremely radiation resistant organism, *D*. *radiodurans* has been extensively studied since several decades to elucidate the molecular mechanisms responsible for its exceptional ability to withstand lethal effects of various DNA-damaging agents, such as ionizing and UV radiation, toxic chemicals and desiccation (for recent reviews, see [1–3]).

Prevalent features playing a key role in this extreme radioresistance have been already described: (i) *D*. *radiodurans* possesses highly proficient DNA double strand break (DSB) repair mechanisms, as homologous recombination (HR) [4], Extended Synthesis-Dependent Strand Annealing (ESDSA) [5,6], and Single-Strand Annealing (SSA) [7,8], that enable *D*. *radiodurans* to accurately reassemble its genome from hundreds of DNA fragments produced by irradiation (ii) *D*. *radiodurans* has also evolved a combination of very efficient non-enzymatic and enzymatic antioxidant defenses which specifically protect proteins against oxidative damage (for reviews, see [3,9,10]) (iii) a highly condensed ring-like nucleoid may also facilitate genome reassembly [11], although this hypothesis is still controversial [9,12].

A rapid and efficient response is required for cell recovery from the various cellular damages induced by irradiation. A subset of *Deinococcus* genus-specific genes, *ddrA*, *ddrB*, *ddrC*, *ddrD*, *ddrI*, *ddrO* (for DNA damage response), and *pprA* (Pleiotropic protein promoting DNA repair), have been identified as strongly induced by exposure to ionizing radiation or desiccation [13]. A common radiation/dessication response motif (RDRM) was found upstream of many highly radiation-induced genes in *D*. *radiodurans* as well as upstream of their homologs in *D*. *geothermalis* and *D*. *deserti*, thus defining the RDR regulon [14]. However, the regulatory mechanisms underlying the response of *D*. *radiodurans* to radiation are still poorly understood. The protein IrrE (also referred to as PprI), was described as a general switch, up-regulating expression of various proteins in *D*. *radiodurans* or *Deinococcus deserti* [15–17]. DdrO was proposed to be the global transcriptional regulator of the RDR regulon [14], and was recently shown to be cleaved in an IrrE-dependent manner upon exposure to ionizing radiation [17].

Finally, it now appears that the extreme radiation resistance of *D*. *radiodurans* is also due to a combination of diverse metabolic and regulatory pathways, but the links making a comprehensive network from these various mechanisms is still missing and several factors acting in these pathways still remain to be discovered.

Large-scale mutant libraries remain an efficient method to identify individual proteins required for a complex biological response such as for radiation resistance. Early efforts were based upon classical MNNG (*N*-methyl-*N’*-nitro-*N*-nitrosoguanidine) mutagenesis technique but this approach identified only a few new *D*. *radiodurans* loci due to the difficulties to map point mutations [18,19]. The transcriptome and proteome approaches have identified differentially regulated genes after exposure to ionizing radiation and desiccation, [13,20–23], but did not allow identification of genes constitutively expressed for cell defense against genotoxic stresses.

Here, we describe a highly efficient *D*. *radiodurans in vivo* mutagenesis method based on a hyperactive version of the Tn*5* transposition system, carried by a temperature-sensitive vector suitable for *D*. *radiodurans* [24] as transposon delivery system. This system combines mutations in the Tn*5* transposase encoding gene (*tnp*) as well as in the ends of the transposon [25–28]. The use of this mutagenesis system provide large collections of mutants since insertions of this element into DNA are highly random and the *in vivo* transposition does not need host factors [29,30]. The *D*. *radiodurans* Tn*5*-based insertion library has been subsequently screened for sensitivity to γ- and UV rays, as well as after exposure to mitomycin C (MMC). The transposon insertion site was mapped on the genome by arbitrary PCR and sequencing for each of the 206 mutants sensitive to at least one of these DNA damaging agents, mainly γ-rays. This analysis and further determination of the mutant survival rates after γ-irradiation enabled us to identify 37 genes that significantly contribute to the extreme resistance of *D*. *radiodurans* to genotoxic stresses. These include genes involved in DNA repair, stress responses and various metabolic processes. We also performed an initial characterization of three previously undescribed radiosensitive mutants inactivated for loci *DR0007*, *DR0265* and *DR2462*.

## Results and Discussion

### Construction of a Tn*5*-based transposon mutant library in *D*. *radiodurans*

To generate a collection of mutants in *D*. *radiodurans*, we have developed an *in vivo* Tn*5*-based mutagenesis system. For this purpose, we cloned a mini-Tn*5* derived (Tn*5*-*hph*) transposable element and a mutant *tnp* gene encoding a hyperactive Tn*5* transposase [26] into a conditionally replicating temperature-sensitive shuttle vector (*repU*Ts), that was shown previously to be stably maintained at 28°C in *D*. *radiodurans* and rapidly lost at 37°C [24] (plasmid p13554, Fig 1A). The Tn*5*-*hph* mini-transposon was constructed by assembling *in vitro* a cassette conferring hygromycin resistance and two flanking optimized 19-bp transposase recognition sequences (Mosaic Ends; [28]) (see Material and Methods for details of the construction). The *tnp* gene encoding the transposase was placed under the control of the P_*spac*_ promoter [31] and cloned outside the mobile element to obtain stable insertions upon the loss of the delivery vector.

**Fig 1:**
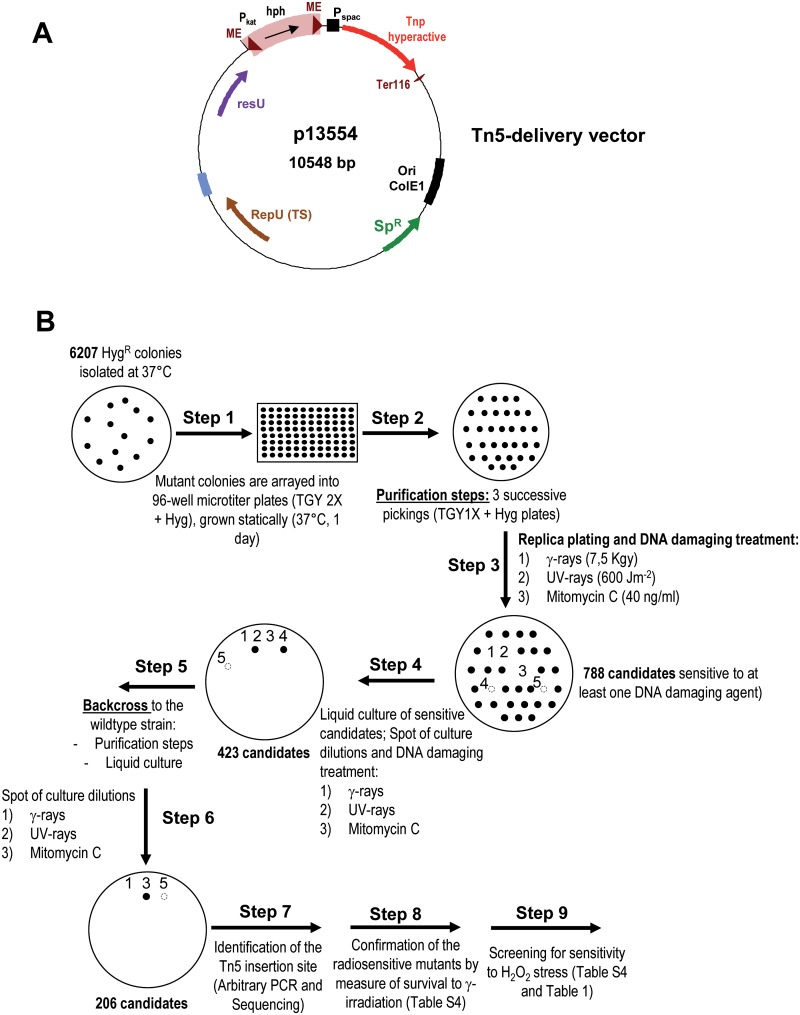
Description of the transposition procedure used to create the *D*. *radiodurans* Tn*5*-insertion mutant library. (A) The Tn*5*-based transposon delivery vector, p13554 is a derivative of the temperature sensitive plasmid p13841 [24]. The mini Tn*5* (Tn*5*-*hph*) consists of a hygromycin resistance cassette (*hph*) as a selectable marker, flanked by the optimized 19-bp mosaic ends (ME) of Tn*5* [28]. The *hph* gene is expressed under the control of the P_*kat*_ promoter. The hyperactive Tn*5* transposase [25,26,94] is cloned outside the mobile element to generate stable insertions and is expressed from the P_*spac*_ promoter inducible with IPTG [31]. Ter116 is a *D*. *radiodurans* transcription terminator. (B) Flow chart of the procedure used to screen the *D*. *radiodurans* Tn*5*-insertion mutant library for sensitivity to DNA damaging agents.

The mutant library was constructed in a *D*. *radiodurans* host (GY10973) expressing the LacI repressor to repress expression of the transposase from the P_*spac*_ promoter and avoid possible toxic effects previously reported in *E*. *coli* [32,33]. The isolation of Tn*5* insertion mutants was performed in two steps: (i) the host strain was transformed with the transposase delivery vector and the transformants were selected at a permissive temperature (30°C) on TGY1X plates supplemented with both hygromycin and spectinomycin. (ii) Transformed cells were cultivated into TGY2X medium at 30°C to an A_650_ ≈ 0.1 (transposition step) and appropriate dilutions were plated on hygromycin plates at the non-permissive temperature (37°C) to simultaneously select for Tn*5*-*hph* insertion mutants and to cure the delivery vector (see Materials and Methods for details). The transposition frequency is about 1x10^-2^ (insertion mutants/viable cell), which is about 10-fold higher than observed in other bacterial species when using the Tn*5* hyperactive transposase [29,30,34], thus demonstrating the efficiency of our method.

To test whether Tn*5*-*hph* insertions occurred randomly into the *D*. *radiodurans* genome, the insertion sites for a sample of 42 hygromycin-resistant mutants isolated at 37°C were localized by an arbitrary PCR procedure followed by DNA sequencing (see Materials and Methods). We found that each Tn*5*-*hph* insertion mapped to a unique location (data not shown), indicating that the insertion mutants of our library were independent and that the insertions occurred randomly throughout the genome of *D*. *radiodurans*.

### Large-scale isolation of ionizing radiation sensitive mutants

A total of 6207 Hyg^R^ single-gene insertion mutants were screened for sensitivity to genotoxic agents such as γ-rays, UV-rays, and MMC according to the flow chart depicted in Fig 1B. The procedure included purification steps (steps 2 and 5 in Fig 1B) followed by two consecutive screening steps, replica plating and semi-quantitative spot test (steps 3 and 4, Fig 1B). Due to the multi-genomic status of *D*. *radiodurans* (4 to 8 genomic copies per cell according to [35]), the purification steps are important to favour homogenotization of the mutated alleles. Mutants that appeared sensitive to at least one DNA damaging treatment after the screening steps were further analyzed by backcross assays (introduction of the insertion into the parental reference strain R1) to confirm the link between the mini-Tn*5* insertion and their respective phenotype (steps 5 and 6, Fig 1B). A cohort of 206 mutants was mapped by arbitrary PCR followed by sequencing and analyzed for their homozygous/heterozygous status by diagnostic PCR (step 7, Fig 1B). Master screening data of backcrossed insertion mutants are given in S1 Table.

As shown in S1 Table, the insertions mapped largely within the coding region of genes except rare events for which the transposon was inserted in intergenic regions. Most of the insertions were found at only one position in a given gene, whereas for around 17% of the mutants, single insertions were found independently at different sites into the same gene (S1 Table). Except the mutants inactivated for *DR0400* and *DRB0002* that were found sensitive to only MMC, the majority of mutants were sensitive to ionizing radiation (IR) and many showed cross-sensitivity to MMC and/or UV. About half of the mutants were heterozygous. These include mutants with insertions in genes involved in essential processes such as DNA replication (*dnaE*, *dnaN*, *holA*), DNA supercoiling (*topA*, *gyrB*), translation and ribosome biogenesis or assembly. Interestingly, the mutant disrupted for *DR2606* encoding the homolog of PriA, a key protein of the main pathway for reactivation of stalled replication fork in bacteria [36,37], is sensitive to γ-, UV-radiation, and MMC. This result suggests the existence of a PriA-dependent replication restart primosome in *D*. *radiodurans* involved in the restoration of an intact genome after irradiation. A classification of the inactivated genes in accordance with the Cluster of Orthologous Groups (COG) data base (S1 Fig) shows that the mutants affected for functions involved in DNA replication, recombination and repair represented only a fraction (8.2%) of the total number of mutants. Indeed, the great majority of mutants were affected in various metabolic pathways including energy, coenzyme, amino acid, nucleotide and lipid metabolism, cell envelope biogenesis, and posttranslational modification (S1 Fig and S1 Table).

To evaluate more precisely the contribution of these genes to radioresistance, we measured the survival of each insertion mutant exposed to γ-rays at high doses of 10 and 15 kGy since sensitivity of repair genes mutants (as *i*.*e radA*, *polX*, *sbcCD*) become apparent only in heavily irradiated cells (for review, see [2]). As shown in S1 Table, the majority of the mutants were only marginally sensitive with less than a log decline in survival after exposure to 15 kGy. Only 37 insertion mutants showed a significant decline in their survival rate (survival rate lower than 6% when exposed to a dose of 15 kGy) as compared to the wild-type strain (Table 1). This result might be partly explained by the different physiological conditions between the screening procedure and the survival assay, which were performed by spot test on TGY plates and in liquid, respectively. The subset of the most radiation sensitive mutants (Table 1) highlights genes involved in diverse DNA repair pathways such as nucleotide excision repair (*uvrA-1*, *uvrB*, and *uvrC*) with a survival rate lower than 1% at 15 kGy, and recombinational repair (*recG*, *recN*, and *ruvA*), all being also sensitive to MMC and at a lesser extent to UV. We also identified *DRA0346* encoding PprA required for accurate cell division following repair of DNA DSB [38–40].

**Table 1:** Genes detected in our screen for which the corresponding *Tn*5 insertion mutants exhibit the highest gamma radiation sensitive phenotype.

Functional category	Mutant locus	survival to γ-rays (%)		Sensitivity to			Comments
		10 kGy	15 kGy	MMC^a^	UV^b^	H_2_O_2_ ^c^	
**CONTROL STRAINS**							
wild-type control		**85**	**38**	R	R	R	
Δ*recA*		**<10** ^**–5**^	**<10** ^**–5**^	SS	SS		
Δ*ddrB*		**1**	**0.1**	S	S		
Δ*polX*		**50**	**5**	s	s		
*katA*::Tn*5*		**48**	**15.5**	R	R	SS	
**DNA replication, recombination, and repair**							
*Nucleotide excision repair*							
UvrA1	*DR1771*	5.7	0.3	SS	s	s	**heterogenote**
UvrB	*DR2275*	6.2	0.7	SS	R	R	
UvrC	*DR1354*	5.0	0.03	S	s	R	
*Homologous recombination*							
RecG	*DR1916*	23.0	4.1	SS	S	R	
RecN	*DR1477*	28.9	5.5	SS	S	R	
RuvA	*DR1274*	18.8	5.4	SS	SS	s	**heterogenote**
*Other mechanisms*							
DNA topoisomerase I	*DR1374*	12.8	1.9	s	s	R	**heterogenote**
PprA	*DRA0346*	0.4	0.1	SS	SS	R	
PriA	*DR2606*	85	4.2	SS	S	R	
**Stress response and other regulatory functions**							
*Radiation resistance*							
Global regulator IrrE	*DR0167*	1	0.01	SS	SS	S	
Response regulator DrRRA	*DR2418*	32	1.5	SS	R	R	
DNA-damage responsive membrane protein	*DR2518*	26.3	6	SS	SS	s	
*Putative transcription factors*							
HTH transcriptional regulator, GntR family (COG2188)	*DR0265*	19.3	0.6	S	S	S	
HTH transcription factor, CAP family DdrI	*DR0997*	4.7	0.4	SS	SS	s	**heterogenote**
**Translation, ribosomal structure and biogenesis**							
30S ribosomal protein S19, RpsS	*DR0315*	39.2	2.7	R	s	s	**heterogenote**
Ribonuclease P protein component (RnpA)	*DR2151*	45.4	0.3	R	R	R	
Alanyl tRNA synthetase, AlaS	*DR2300*	23.2	2.7	s	s	s	**heterogenote**
**Metabolic pathways**							
*Energy production and conversion*							
Rieske like Fe-S protein (COG0723)	*DR0342*	51.2	4.6	s	s	R	
Cytochrome C-Type biogenesis protein CycJ	^(u)^ *DR0347*	4.5	1.2	R	R	R	
Aromatic compound dioxygenase, ferredoxin (COG2146)	*DR1950*	24.5	0.6	S	R	R	**heterogenote**
Cytochrome C oxidase subunit III, CyoB	*DR2620*	39.1	5.3	R	R	NA^d^	**heterogenotegrowth defect**
Malic enzyme	^(o)^ *DRA0276*	39.4	4.2	R	R	R	
*Carbohydrate metabolism*							
Fructokinase RbsK ortholog	*DR1525*	48.7	3.4	SS	S	s	
*Amino acid transport and metabolism*							
Glycine/serine hydroxymethyltransferase (GlyA)	*DR0038*	41	2.1	s	s	R	
*Metabolism of coenzymes*, *cofactors and vitamins*							
Pantothenate synthetase, PanC	*DR1164*	51.6	4.1	S	S	R	
ApbE family protein (involved in thiamine biosynthesis)	*DR1794*	42.4	5.6	R	R	R	**heterogenote**
Ketopantoate hydroxymethyl transferase	*DR2615*	44.5	4.3	SS	S	R	
*Membrane transport*							
Signal peptidase I (COG0681)	*DR1321*	42.6	4.6	S	S	R	
NRAMP family membrane transporter	*DR1709*	28.8	4.6	R	s	R	**heterogenote**
**Poorly characterized or uncharacterized proteins**							
Uncharacterized conserved protein (COG1624)	*DR0007*	10.1	0.5	SS	s	R	
Small nucleotidyltransferase- like protein (COG1669)	*DR0679*	28.3	1.4	s	s	S	
Exopolyphosphatase-related protein (COG0618) or DHH superfamily hydrolase	*DR0826*	33.1	2.5	SS	s	s	
Protein containing an N-terminal CDNR domain	*DR1740*	38.5	5.5	R	R	NA^d^	**heterogenotegrowth defect**
Uncharacterized secreted protein	*DR2058*	51.9	4.6	R	R	R	
RNase Y family	*DR2462*	30.1	0.9	S	SS	R	
Predicted membrane protein	*DR2572*	38.9	3.4	R	s	R	
Uncharacterized conserved secreted enzyme	*DRA0022*	40.5	4.8	R	R	R	**heterogenote**
intergenic insertion	between *DR0251* and *DR0252*	24.2	0.1	R	R	R	
intergenic insertion	between *DR2613* and *DR2614*	33.2	5.3	R	R	R	

^a, b^SS, highly sensitive, survival <10^-6^; S, sensitive, survival comprised between 10^-6^ and 10^-4^; s, slightly sensitive, survival comprised between 10^-4^ and 10^-2^; R, resistant, survival >10^-2^.

^a^ onto TGY plates supplemented with 40 ng/mL mitomycin C (see Materials and Methods).

^b^ onto TGY plates exposed to 600 J m^-2^ UV-rays (see Materials and Methods).

^c^ by using the disc inhibition assay as described in Materials and Methods and Fig 2 (for the inhibition diameters corresponding to the different levels of sensitivity).

^d^ NA: Not applicable because of growth defect.

^(o)^ gene is located in a putative operon (predicted by FGENESB (www.softberry.com).

^(u)^ transposon inserted in the upstream region of the corresponding CDS.

The presence of genes involved in DNA repair and stress response regulation among the genes listed in Table 1 was expected. However, several genes, such as the *recA*, *recO* and *recR* genes involved in DNA double strand break repair by ESDSA and homologous recombination [41], or more generally known for their involvement in radioresistance, were not found by our screening procedure. Previous work showed that a limited number of RecA molecules (2500 molecules per cell in place of 44000 RecA molecules per cell in the wild type) were sufficient to ensure the same survival as those of the wild type bacteria upon γ-irradiation [42]. Interestingly, we found a slightly radiosensitive *recF* mutant in our screening on plates and we showed that mutation was not homogenotized. The purification steps on selective medium to favor homogenotization of the insertion mutations were not sufficient to obtain homogenotes when the mutations conferred an important selective disadvantage (S1 Table). We verified by PCR the homogenotization status of the mutants and 100 over 206 were found heterozygous, some of them being essential genes involved in DNA replication, DNA supercoiling, translation or metabolism (see S1 Table). Our screening also did not uncover the non-essential *ddrA* and *ddrB* genes, known to be involved in protection of 3’ single-stranded DNA ends and in DNA DSB repair by single-strand annealing, respectively. Given that 14957 independent mutants are required to obtain a 99% chance of inactivating a particular gene in *D*. *radiodurans* ([43] and see Material and Methods), we did not expect to identify all the genes involved in radioresistance. Indeed, we can estimate that approximately 14% of the genes were not inactivated among the 6207 insertion mutants we screened (see Material and Methods for calculation details).

Our screening also highlights genes previously reported as key players in the regulation of the radiation/dessication response of *D*. *radiodurans* such as: (i) *irrE* encoding the positive regulator of the DNA damage response [15–17,44]; (ii) *DR2418* encoding DrRRA, the response regulator of a two-component system responsible for transcriptional regulation of numerous genes related to stress response and DNA repair [45]; (iii) *DR2518* encoding a DNA damage-inducible membrane protein kinase important for DNA DSB repair [46,47]. In addition, we also isolated the heterozygous insertion mutant *ddrI* (*DR0997*), a DNA damage response gene encoding a transcription factor of the CAP family whose relative expression was shown to be significantly reduced in the *drRRA* mutant under both normal conditions and γ-radiation stress [45].

The main category of radiation sensitive mutants isolated in this study affects various metabolic functions. These results agree with recent data indicating that metabolic reprogramming plays a central role in the survival of organisms exposed to oxidative stress [48]. Mn ions play a major role in protecting proteins from oxidative damage as component of non-enzymatic metabolite complexes [49]. In accordance with the importance of a high Mn/Fe ratio for efficient recovery from irradiation injury, we isolated two mutants likely impaired in this ratio: (1) one heterozygous mutant for the gene *DR1709*, encoding a NRAMP family Mn(II) transporter [14], in which the intracellular concentration of Mn^2+^ might be lower than in wild type (2) the second mutant was inactivated for *DR2106* encoding SufB (S1 Table), a member of the SUF system involved in both assembly and repair of oxidized oxygen-labile Fe-S clusters [50]; for a review, see [51]. Thus, alteration of SufB activity may increase intracellular free iron, promoting oxidative damage through the Fenton reaction [9,52]. SufB was also shown upregulated in *D*. *geothermalis* aerobically cultivated in low-Mn medium [53]. Likewise, inactivation of *DR1321* encoding signal peptidase I protein might affect the release of exogenous amino acids and peptides. These products are important to protect cellular proteins against oxidative damage as components of manganese complexes and/or membrane proteins required for metabolite transport or cell wall integrity [49]. Disruption of gene *DRA0276*, encoding the malic enzyme that converts malate to oxaloacetate in the glyoxylate bypass and TCA cycle, might also contribute to lower the *D*. *radiodurans* capacity to deal with ROS generated by γ-irradiation. The malic enzyme has been shown to be involved in the NADH-to-NADPH conversion cycle used by *Pseudomonas fluorescens* to counteract oxidative stress [48].

A significant number of radiosensitive mutants were inactivated for genes involved in various metabolic functions such as energy production and conversion, amino acid transport and metabolism of coenzymes and cofactors (Table 1). In particular, inactivation of *DR0342*, *DR0347*, *DR1950*, *DR2620* genes will greatly impair electron transfer through the respiratory chain as well as the associated ATP production. A failure to respond to the increased demand of energy during cell recovery could explain the IR sensitivity of these mutants. Likewise, protein synthesis which is essential for cell survival after irradiation [54] might be disturbed in another class of IR sensitive mutants such as *DR0315*, *DR2151*, and *DR2300* affected in translation activity.

**Fig 2:**
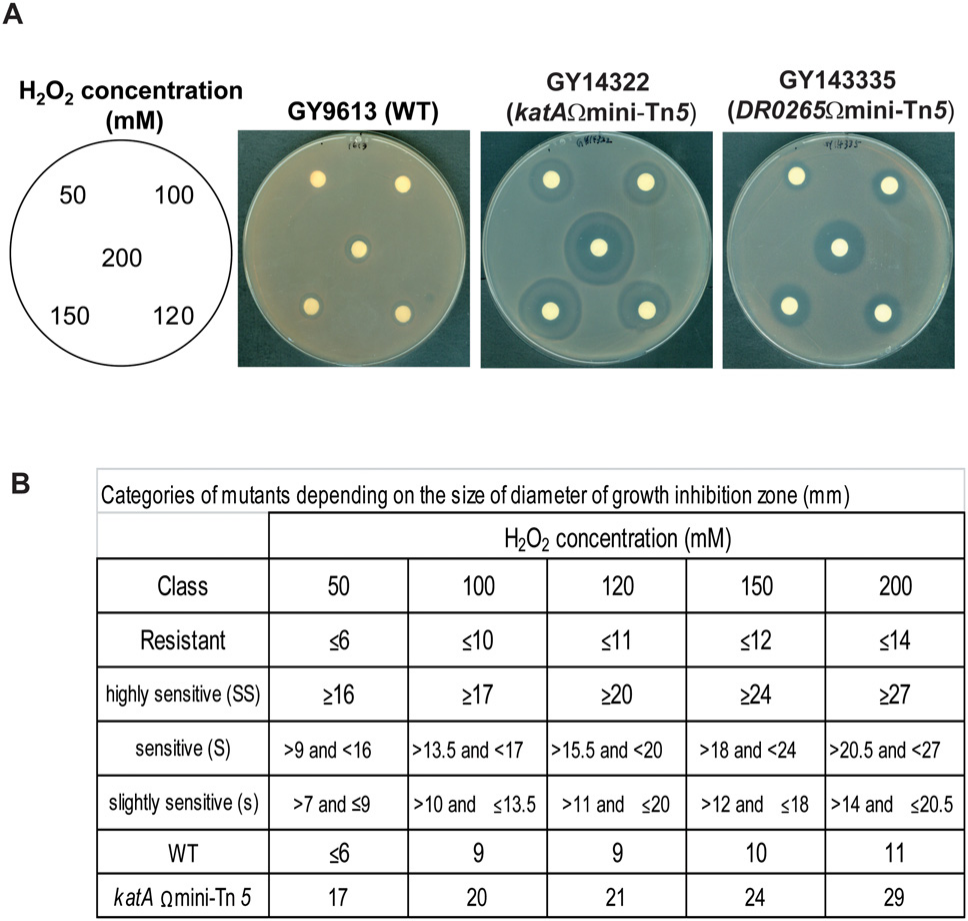
Measurement of sensitivity to H_2_O_2_ stress by disc inhibition assay (procedure described in Materials and Methods). (A) Phenotype of the wild-type (resistant), the *katA* mutant (highly sensitive), and the *DR0265* mutant (middle sensitive) are shown. (B) The mutants are classified into four categories depending on the diameter (in mm) of growth inhibition area as indicated.

Given the γ-radiation-induced production of intracellular ROS, we assessed whether the observed radiosensitive phenotype was primarily due to ROS sensitivity or not, by testing 158 mutants for their sensitivity to hydrogen peroxide stress (Table 1 and S1 Table) as described in Material and Methods and Fig 2. The few mutants found sensitive to oxidative stress fall into two categories: (i) the expected one, including the *katA* mutant (Table 1 and Fig 2; [55,56]), the *irrE* mutant, previously shown to exhibit significantly reduced catalase activities [57], and the mutant inactivated for *DR2518* encoding a DNA-damage sensor kinase (RqkA) involved in DNA repair and whose kinase activity is stimulated *in vitro* by the antioxidant pyrroloquinoline-quinine (PQQ) [46] (ii) the previously undescribed mutants including regulatory mutants inactivated for the transcription factors DR0265 (Fig 2) and DdrI (Table 1), and mutant for *DR0679*, encoding a putative small nucleotidyltransferase (Table 1). In addition, several less γ-rays sensitive mutants were found sensitive to hydrogen peroxide. These include the *DR1131* mutant, inactivated for the *hemZ* gene involved in heme biosynthesis, the mutant for *DR1207* of unknown function, the *DR2374* mutant, inactivated for a ribonucleotide reductase of archeal type, and the mutant for *DR2417m*, encoding DncA, a novel essential β-CASP family nuclease contributing to the radiation resistance of *D*. *radiodurans* ([58] and S1 Table). The low fraction of oxidative stress mutants identified in our library may also be due to the non-viable status of the Tn*5*-insertions into the genes essential for oxidative stress recovery in response to high level of radiations.

This broad distribution of the inactivated genes among diverse functional categories confirms that multiple pathways participate to the extraordinary radiation resistance of *D*. *radiodurans*. In addition to known genes involved in repair of DNA damage or in regulation, we have identified new genes whose involvement in IR resistance was not previously reported or suspected. Inactivation of six of these genes, resulted in a highly γ-radiation sensitive phenotype (with a survival to γ-rays of <1% at 15 kGy; Table 1). Among these six genes, we targeted the two putative *D*. *radiodurans* loci *DR0007* (encoding a di-adenylate cyclase homolog) and *DR2462* (encoding an RNase Y homolog), as well as *DR0265* (likely encoding a transcription factor of unknown function). A more complete functional characterization of knockout mutants of these three genes is described below.

### Cyclic di-AMP (c-di-AMP) contributes to radioresistance of *D*. *radiodurans*

One of the radiosensitive mutants previously undescribed was inactivated for the *DR0007* locus. This gene encodes a homolog of CdaA (37% amino acid identity with *Bacillus subtilis* YbbP, renamed CdaA), one of the three diadenylyl cyclase (DAC) enzymes found in *B*. *subtilis*, that catalyzes signalling nucleotide c-di-AMP synthesis [59]. The second gene, *DR0008* encodes a homolog of CdaR, which stimulates the diadenylate cyclase activity of CdaA in *B*. *subtilis* [59]. Sequence analysis of this locus with FGENESB (www.softberry.com; [60]) suggests that *DR0007*, *DR0008* and *DR0009* are within a predicted operon. Accordingly, these three genes showed highly significant correlation of their expression pattern, based on transcriptome analysis [20].

**Fig 3:**
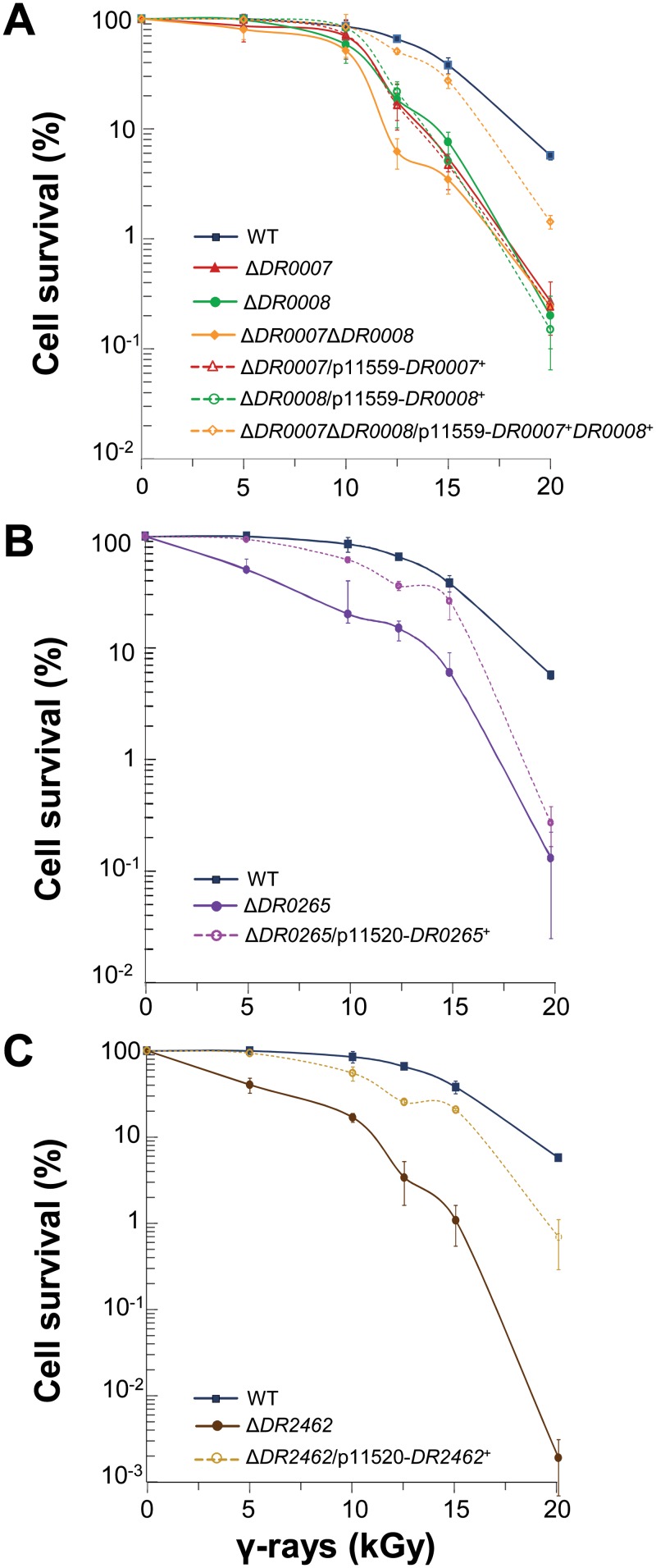
The *D*. *radiodurans* mutants deleted for *DR0007*, *DR0008* or both (A), *DR0265* (B) and *DR2462* (C) show increased sensitivity to γ-irradiation. Bacteria were exposed to γ-irradiation at doses indicated on the abscissa. Symbols: (A) wild-type (blue squares), Δ*DR0007* (red closed triangles), Δ*DR0008* (green closed circles), Δ*DR0007*Δ*DR0008* (yellow closed diamonds), Δ*DR0007*/p11559-*DR0007* ^+^ (red open triangles), Δ*DR0008*/p11559-*DR0008* ^+^ (green open circles), Δ*DR0007* Δ*DR0008*/p11559-*DR0007* ^+^ *DR0008* ^+^ (yellow open diamonds). (B) wild-type (blue squares), Δ*DR0265* (purple closed triangles), Δ*DR0265*/p11520-*DR0265* ^+^ (pink open triangles). (C) wild-type (blue squares), Δ*DR2462* (brown closed circles), Δ*DR2462*/p11520-*DR2462* ^+^ (ochre open circles).

**Fig 4:**
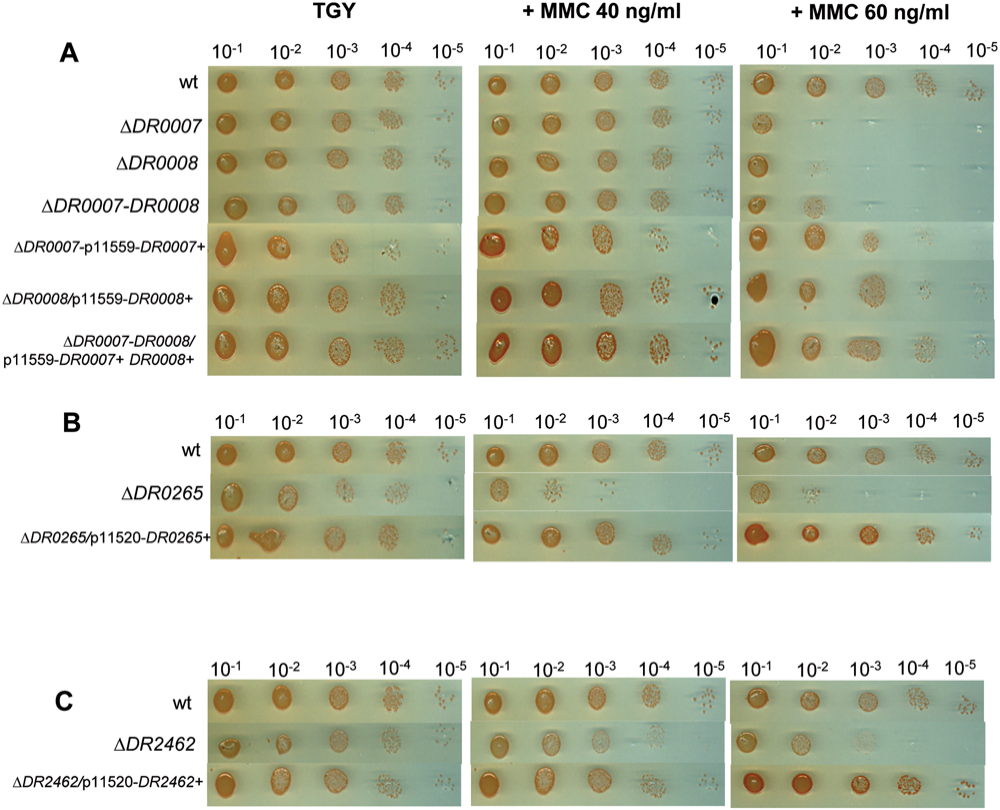
Deletion of *DR0007*, *DR0008* or both (A), *DR0265* (B), or *DR2462* (C) sensitizes *D*. *radiodurans* to MMC. Bacteria were grown in TGY2X liquid medium to A_650_ = 1, serially diluted and dilutions were spotted onto TGY agar plates supplemented or not with MMC at the indicated doses, and supplemented with spectinomycin for strains harboring derivatives of p11559 or p11520 plasmids.

**Fig 5:**
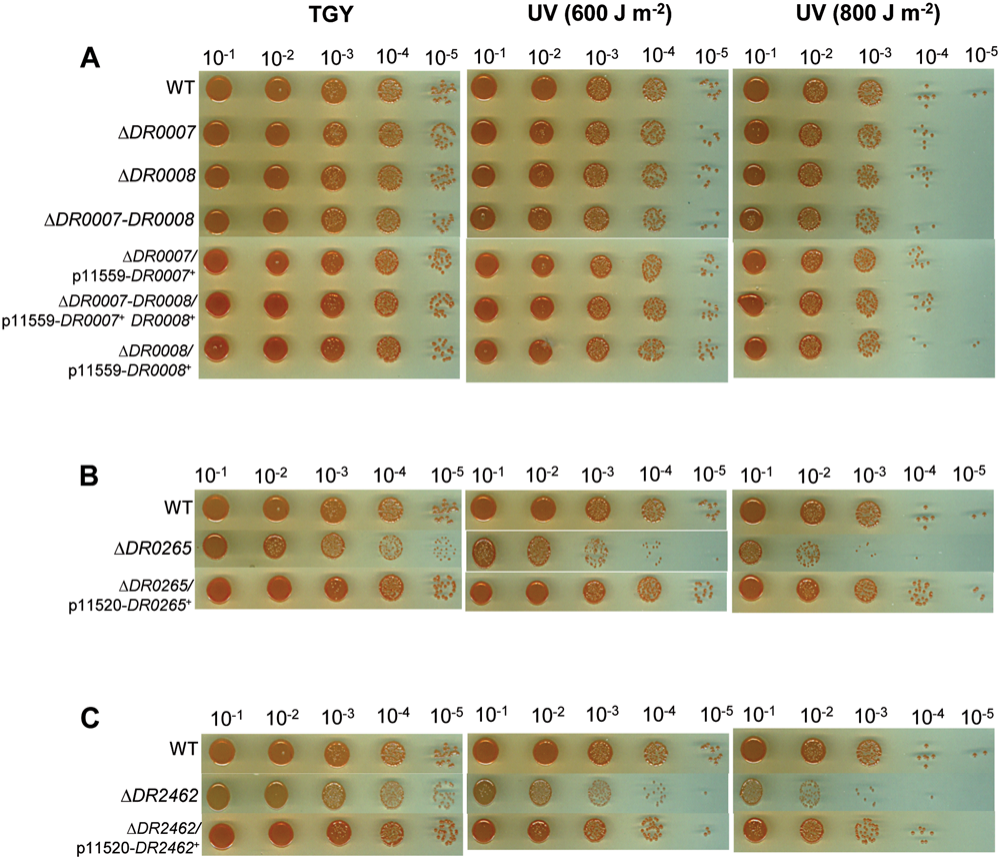
Δ*DR0265* and Δ*DR2462* are sensitive to UV but not Δ*DR0007* and Δ*DR0008*. Bacteria were grown in TGY2X liquid medium to A_650_ = 1, serially diluted and dilutions were spotted onto TGY agar plates subsequently exposed to UV-irradiation at the indicated doses.

To confirm the importance of these proteins for *D*. *radiodurans* recovery after irradiation, several single and double deletion mutants were constructed. Homogenotes of the Δ*DR0007* mutant were easily obtained on selective medium and did not display any growth defect under standard cultivation conditions (same doubling time as those of the wild type R1 strain), indicating that *DR0007* gene is not essential for cell viability under our culture conditions. The survival of Δ*DR0007* was decreased by a factor of 7- and 21-fold after γ-irradiation at 15 and 20 kGy, respectively, when compared to the wild type strain. Ectopic expression of *DR0007* gene alone did not fully restore the wild type radiation resistance (Fig 3A). Although we did not find insertions in *DR0008* in our initial screening, we constructed a *DR0008* deletion mutant to completely inactivate the gene. The Δ*DR0008* mutant displayed the same γ-radiation sensitive phenotype as the Δ*DR0007* mutant, and again ectopic expression of *DR0008* did not restore the wild type phenotype (Fig 3A). The double mutant Δ*DR0007* Δ*DR0008* showed the same survival rate than those of the single mutants (Fig 3A). In contrast, ectopic expression of both of these genes in the double mutant was sufficient to restore the wild type radiation resistance (Fig 3A). These results suggest that *DR0007* and *DR0008* are functionally linked and that a coordinated expression of the two genes is required to fulfill their role in radioresistance. To examine whether c-di-AMP could complement the radiosensitive phenotype of Δ*DR0007* and Δ*DR0008*, exogenous c-di-AMP was added immediately after exposure to γ-radiation, in the presence of polyamines to favor c-di-AMP uptake according to the procedure of Oppenheimer-Shaanan *et al*. [61]. Nevertheless, addition of c-di-AMP did not restore radiation resistance in the single and double mutants. This may be due to the use of polyamines which decrease cell survival even in the wild type (by a factor of 3.5 at 10 kGy and 4 at 15 kGy). Finally, deletion of *DR0007*, *DR0008* or both only slightly sensitizes the cells to MMC (Fig 4A) and to UV light (Fig 5A). Deletion of *DR0009* gene did not affect resistance to γ-irradiation, but we cannot exclude that *DR0025* that shares 35% identity and 53% similarity with *DR0009* may functionally complement the deletion of *DR0009*. This putative redundancy of function may explain why we did not find insertion mutants inactivated for *DR0009* in our initial screening.

To determine whether the increased sensitivity to ionizing radiation of the Δ*DR0007* mutant was due to an altered DNA repair capacity after irradiation, we measured by pulse-field gel electrophoresis the kinetics of DNA DSB repair in the Δ*DR0007* mutant exposed to 3.8 kGy γ-irradiation. The Δ*DR0007* mutant reassembles an intact genome as fast as the wild type strain after exposure to γ-irradiation, indicating that its radiosensitivity is unrelated to a defect in DNA DSB repair.

Interestingly, identification, in our screening for radiosensitive mutants, of a putative c-di-AMP synthesizing enzyme, the DR0007 protein, and its potential positive regulator, the DR0008 protein suggests the contribution of c-di-AMP signalling in the extreme radiation resistance of *D*. *radiodurans*. c-di-AMP is a second messenger recently discovered in bacteria, and involved in the control of diverse cellular pathways (for reviews, see [62–64]) such as regulation of fatty acid synthesis [65], response to cell wall stress [66–68], regulation of potassium transport [63,69]. In addition, the common riboswitch class *ydaO* has been recently identified as receptors for c-di-AMP to control different biological processes [70]. As most of the bacterial species, *D*. *radiodurans* encodes a single DAC (diadenylate cyclase) domain-containing protein, the DR0007 product, while *B*. *subtilis* harbors three specialized c-di-AMP synthases: DisA (DNA Integrity Scanning protein) which is involved in coupling DNA integrity with progression of sporulation [61,71], CdaA and CdaS (which is sporulation-specific). In contrast to DisA, the DR0007 protein does not contain any DNA binding domain. Although we showed that both DR0007 and DR0008 proteins are required for radioresistance, the specific set of receptor and effector proteins of the c-di-AMP signaling system in *D*. *radiodurans* remain to be identified.

### Identification of a new transcription factor, DR0265 involved in extreme radioresistance of *D*. *radiodurans*

Our screen also identified a new radiosensitive mutant inactivated for *DR0265*, encoding a putative transcription factor belonging to the GntR family. This protein contains the C-terminal effector-binding domain UTRA (UbiC transcription Regulator Associated domain) of the HutC subfamily [72]. While the repertoire of HTH-containing proteins identified in *D*. *radiodurans* reflects the diversity of prokaryotic transcriptional regulators [73], only two members of the HutC subfamily (*DR0265* and *DRA0211*) are encoded by *D*. *radiodurans* (versus 24 HutC-like regulators in *Streptomyces coelicolor*, 6 in *E*. *coli* and 7 in *B*. *subtilis*).

Compilation of palindromic *cis*-acting elements recognized by regulators of the GntR family has identified the sequence 5’GT-N(1)-TA-N(1)-AC 3’ as the *cis*-element consensus for the HutC subfamily [72]. Members of this subfamily control various biological processes (antibiotic production, sensing of nutritional status, growth, proliferation and development). In *Pseudomonads*, *Klebsiella* and *Brucella*, HutC acts as a transcriptional repressor of the Hut system responsible for histidine utilization (for review, see [74]). In *D*. *radiodurans*, the Hut operon (*DRA0151*–*DRA0147*) is preceded by the RDRM sequence, and therefore may belong to the predicted radiation response regulon [14]. Nevertheless, it is unlikely that transcriptional regulation of the *hut* operon of *D*. *radiodurans* is mediated by a HutC-type factor for the following reasons: (i) the consensus sequences for HutC subfamily are not found upstream of the *D*. *radiodurans hut* operon. (ii) the organization of the *D*. *radiodurans hut* operon shows similarity to those of *Corynebacterium resistens* with an adjacent gene encoding a transcription regulator of the IclR family (*DRA0152*), and this IclR factor has been shown to activate the *hut* operon in *C*. *resistens* [75], suggesting a similar regulation of the Hut system in both organisms.

Deletion of *DR0265* sensitizes cells to γ-irradiation, as shown by the 7- and 44-fold decrease of Δ*DR0265* survival at doses of 15 and 20 kGy, respectively, when compared to those of the wild type strain (Fig 3B). *Trans* expression of the *DR0265* gene under control of its own promoter restored the γ-ray resistance of the mutant strain to the wild type level (Fig 3B), confirming that the radiosensitivity of Δ*DR0265* was solely due to the absence of the DR0265 protein. Δ*DR0265* bacteria were also sensitive to UV light (Fig 5B), but only slightly sensitive to MMC (Fig 4B). As previously observed with Δ*DR0007*, the kinetics of DNA DSB repair of Δ*DR0265* mutant shows no delay in restoration of intact genomic DNA after γ-irradiation compared to the wild type. Interestingly, Δ*DR0265* bacteria were shown to be among the rare mutants found sensitive to hydrogen peroxide (Table 1, Fig 2), suggesting that *DR0265* may be involved in the response to ROS but the targets of this putative regulator remain to be discovered.

### Involvement of a putative RNase Y in *D*. *radiodurans* extreme radioresistance

Another gene identified as important for radioresistance, *DR2462*, encodes a homolog of the RNase Y recently discovered in *B*. *subtilis* [76–78], as a key endoribonuclease for mRNA turnover, with an important general role in synthesis of components involved in DNA replication, iron metabolism and the cell envelope and cell wall [79,80].

To confirm the role of the DR2462 protein in the *D*. *radiodurans* radioresistance, we have constructed a Δ*DR2462* mutant. Homogenotes of the Δ*DR2462* mutant were easily obtained. Bacteria devoid of the DR2462 protein were sensitive to γ-irradiation as shown by the strong decrease of their survival rate compared to those of the wild type (38-fold and 3021-fold decrease at 15 and 20 kGy, respectively) (Fig 3C). They were also moderately sensitive to UV light (Fig 5C) while they showed a wild type resistance to MMC (Fig 4C). To prove that the absence of the DR2462 protein is solely responsible for the γ-ray sensitivity, the *DR2462* gene including its natural promoter and its putative transcription terminator was cloned into plasmid p11520 and the resulting plasmid p13563 was used to transform Δ*DR2462* bacteria. The resulting transformants recovered the typical wild type survival following exposure to γ-rays, even at a massive dose of 20 KGy, confirming the requirement of *DR2462* for the radiation resistance (Fig 3C).

**Fig 6:**
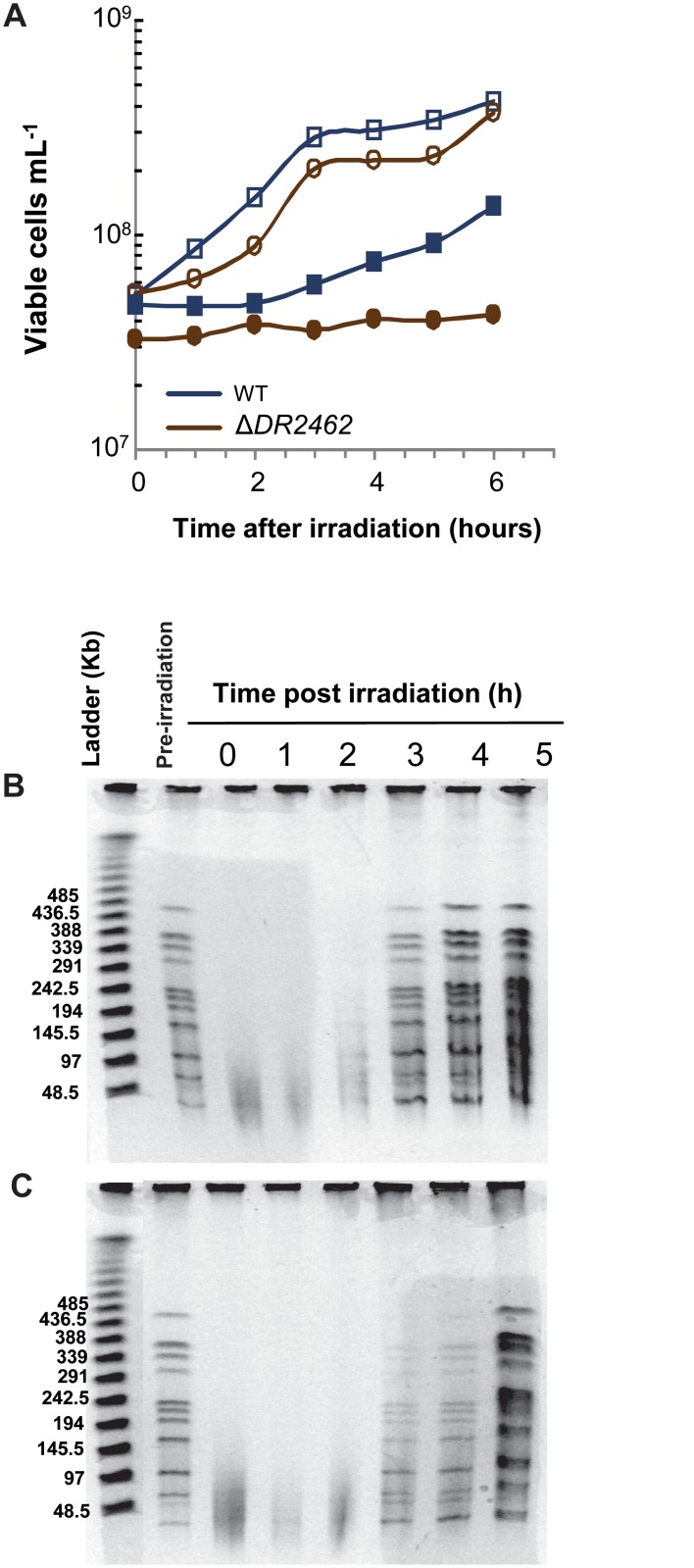
The Δ*DR2462* bacteria show an increased delay in cell division and in reconstitution of genomic DNA after γ-irradiation. A. Growth delay after irradiation. Wild type (blue squares) and Δ*DR2462* (brown circles) bacteria were exposed (filled symbols) or not (open symbols) to γ-irradiation at a dose of 3.8 kGy, diluted in TGY2X to an A_650_ of 0.3 and incubated at 30°C. At different times after irradiation, aliquots were taken to measure the number of viable cells per mL. B and C. Kinetics of restoration of genomic DNA. Bacteria were treated as in (A). DNA agarose plugs were prepared at the indicated post-irradiation times and digested with *Not*I prior to analyses by PFGE. B: wild type; C: Δ*DR2462*.

The kinetics of DNA DSB repair in the Δ*DR2462* mutant exposed to 3.8 kGy γ-irradiation was measured by pulse-field gel electrophoresis. Cells devoid of DR2462 protein showed a delay shorter than one hour in the restoration of an intact genome after irradiation compared to the wild type bacteria (Fig 6B) but it seems that replication did not restart immediately after reconstitution of the genome (Fig 6B), a delay that was also observed for cell division restart (Fig 6A).

**Fig 7:**
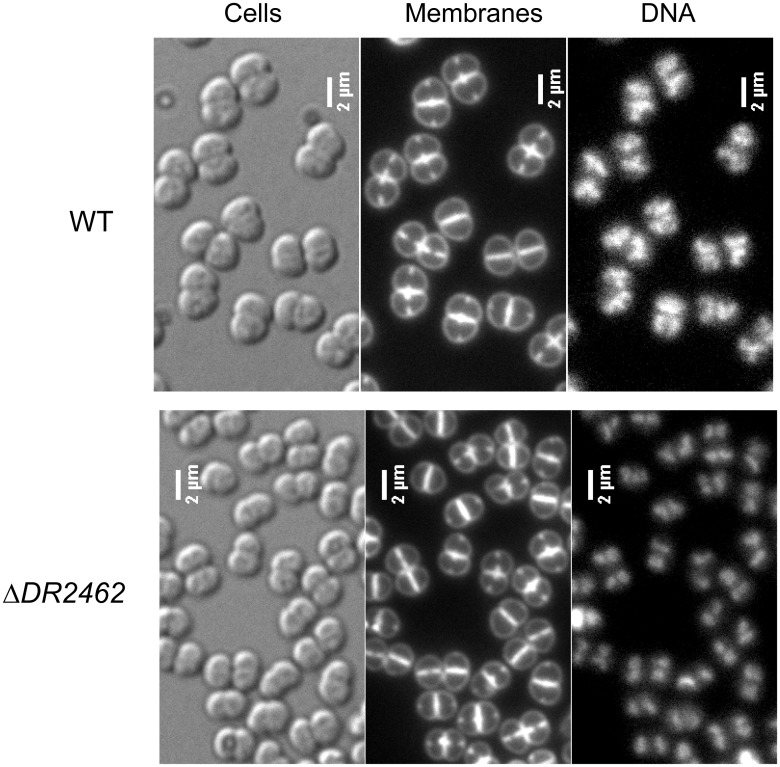
Cell morphology in *D*. *radiodurans* deleted for *DR2462* observed by microscopy. *D*. *radiodurans* cells from the wild type strain (top panels) and from Δ*DR2462* strain (bottom panels) were grown to OD_650_ = 0.3. Left panels: Nomarski interference contrast (DIC). Middle panels: membrane staining (FM4-64). Right panels: DNA staining (DAPI). All pictures are at the same scale (bar = 2 μm).

In *B*. *subtilis*, depletion of RNase Y (YmdA) resulted in an aberrant distribution of cell lengths, with a few unusually longer cells and many short, almost spherical, cells reminiscent of minicells [81,82]. It was proposed that this phenotype might be related to increased concentration of *dnaA* transcript [80], since overexpression of DnaA has been linked to aberrant changes in cell shape [83]. *D*. *radiodurans* mutant bacteria devoid of DR2462 protein grew normally and did not show dramatic alterations of their morphologies when they were observed by microscopy after nucleoid and membrane staining, except that they are slightly smaller than wild type cells (Fig 7), suggesting a more modest role of RNase Y in *D*. *radiodurans* cell viability than those played by RNase Y in *B*. *subtilis*.

## Conclusion

As indicated by [84], broad genetic screens to identify all processes contributing to radiation resistance are very difficult to perform in *Deinococcus*, due to its multi-genomic status. For these reasons, they decided to identify genes involved in radioresistance in an *E*. *coli* strain exhibiting levels of radiation resistance approaching that of *D*. *radiodurans* [85]. They used a Transposon Directed Insertion Sequencing (traDIS) strategy to locate the transposon insertion and to compare the frequency of each insertion within an un-treated population and a population subjected to repeated exposures to ionizing radiation. They identified 46 candidate genes that appear to have a significant role in survival after exposure to ionizing radiation [84]. Here, using a more classical strategy taking into account the multi-genomic status of *D*. *radiodurans*, we identified 37 genes and two intergenic sequences highlighting the importance of DNA repair, stress response, translation but also energy production, carbohydrate metabolism, membrane transport and poorly characterized or uncharacterized proteins in *Deinococcus* radioresistance. Among the 46 genes identified as involved in *E*. *coli* radioresistance by Byrne et al ([84]), we found 9 *D*. *radiodurans* homologs involved in DNA metabolism *(recN*, *recG*, *recR*, *recF*, *uvrA*, *uvrB*, *uvrC*, *topA*) or with uncharacterized functions (*DR1167* homolog of the *E*. *coli yabA*). We also found an insertion located in the intergenic region between *DR2614* and *DR2613* (the latter encoding a homolog of the *E*. *coli yab1* gene). Interestingly, we found the key genes involved in regulation of the DNA damage response, most being specific to the *Deinococcaceae*. We more extensively characterized three mutants not found in E. coli as contributing to radioresistance, impaired in a putative transcriptional regulator, a putative protein of the RNase Y family and a putative protein proposed to be involved in c-di-AMP synthesis, all being never described to date to participate to *Deinococcus* radioresistance. All the genes identified in *E*. *coli* [84] and, here in *D*. *radiodurans*, as participating to radioresistance reinforce the idea that a complex network involving efficient DNA repair, protein protection against oxidation, and tightly coordinated combination of diverse metabolic and regulatory pathways, is the key of bacterial radioresistance.

## Materials and Methods

### Bacterial strains, media and growth conditions

Bacterial strains are listed in S2 Table. *E*. *coli* strain DH5α was the general cloning host and strain SCS110 was used to propagate plasmids prior to introduction into *D*. *radiodurans* via transformation [86]. To produce p13554 in *E*. *coli*, we used XL1Blue that expresses *lacI* ^*Q*^ on the F’ plasmid to avoid the toxicity due to overproduction of Tn*5* transposase as described previously [32]. All *D*. *radiodurans* strains were derivatives of strain R1 ATCC 13939. TGY2X liquid medium and TGY plates [87] were used for *D*. *radiodurans* growth and Luria-Bertani (LB) broth for *E*. *coli*. Media were supplemented with the appropriate antibiotics used at the following concentrations: spectinomycin 40 μg mL^-1^ for *E*. *coli* and 75 μg mL^-1^ for *D*. *radiodurans*; hygromycin 50 μg mL^-1^ for *E*. *coli* and 50 to 100 μg mL^-1^ for *D*. *radiodurans*, kanamycin 25 μg mL^-1^ for *E*. *coli* and 6 μg mL^-1^ for *D*. *radiodurans*. When necessary, expression of Tn*5* transposase was induced by adding 1 mM IPTG in media. Transformation of *D*. *radiodurans* with genomic DNA, PCR products, or plasmid DNA was performed as previously described [87].

### Construction of deletion mutants in *D*. *radiodurans*

To construct each mutant deleted for a given gene, the locus of interest was replaced with the appropriate antibiotic resistance cassette (either the Hyg^R^ resistance cassette expressed from the P_*kat*_ promoter or the Tet^R^ resistance cartridge expressed from the P_groEL_ promoter) using the tripartite ligation method [88]. The deletion mutants generated in this way and used in this study are: Δ*pprA*Ω*hph* (for use as a control mutant in treatment with DNA damaging agents), Δ*DR0007*Ω*hph*, Δ*DR0008*Ω*hph*, double mutant Δ*DR0007*–*DR0008*Ω*hph*, Δ*DR0009*Ω*hph*, Δ*DR0265*Ω*hph*, Δ*oxyR*Ω*hph*, Δ*oxyR2*Ω*tetA* (for using as control mutants in screening for sensitivity to H_2_O_2_) and Δ*DR2462*Ω*hph*. The double mutant Δ*oxyR*Ω*hph* Δ*oxyR2*Ω*tetA* was constructed by transforming the Δ*oxyR2*Ω*tetA* single mutant with genomic DNA from the Δ*oxyR*Ω*hph* mutant. See S3 Table for oligonucleotides used for strain construction. The genetic structure and the purity of the resulting mutant strains were verified by PCR. Oligonucleotides used for diagnostic PCR and sequencing are available upon request.

### DNA manipulations

Plasmid DNA was extracted from *E*. *coli* using the NucleoSpin Plasmid miniprep kit (Macherey-Nagel). *D*. *radiodurans* chromosomal DNA was isolated as described previously [89]. PCR reactions were carried out with Phusion DNA Polymerase (Thermo Scientific) to amplify fragments subsequently used for cloning or with GoTaq Flexi DNA Polymerase (Promega) for all other applications. PCR products were purified using the NucleoSpin Gel and PCR Clean-up kit (Macherey-Nagel). To analyze the homozygous/heterozygous status of the mutants, diagnostic PCR were performed using appropriate pairs of primers encompassing the Tn*5* insertion site. Oligonucleotides used for all these diagnostic PCR are available upon request.

### Construction of Tn*5* delivery vector, p13554

The *lacI* ^*q*^ gene was amplified by PCR using primers ForLacI and RevLacIBg and the plasmid pTRC99A (Pharmacia, see also S2 Table) as template. The PCR fragment was digested by *Bgl*II, and ligated into the *Bam*HI site of p13841, resulting in the plasmid p13537 expressing *lacI* ^*q*^ in the reverse orientation to P_*spac*_. The *tnp* gene encoding the hyperactive Tn*5* transposase was amplified by PCR with primers Tnp5UP and Tnp5Dra and plasmid pWH1891 ([90]; see also S2 Table) as template. After cleavage with *Nde*I and *Dra*I, the PCR fragment was ligated into plasmid p13537, generating plasmid p13545, expressing the transposase under the control of P_*spac*_. The mini-Tn*5*-Hyg^R^ transposon was then amplified by PCR with primers MEUpBst and MEDnBgl and plasmid p12625 as template. After digestion with *Bst*1107I and *Bgl*II, the PCR fragment containing ME (mosaic ends derived from Tn*5*) was inserted into p13545 to generate plasmid p13547.

To use the Tn*5*-delivery system into *D*. *radiodurans* GY10973 strain expressing an additional chromosomal copy of *lacI* under the control of P_*tufA*_ promoter, we have deleted *lacI* ^*q*^ from p13547 as follows: p13547 was digested with *Dra*I and *Psc*I and the 10531-bp fragment was ligated to a linker containing the *Dra*I and *Psc*I ends and an internal *Sal*I site, to generate plasmid p13554 (see S3 Table for oligonucleotides used for p13554 construction and Fig 1).

### Plasmids used for complementation analyses

Please see S2 and S3 Tables for details of construction of plasmids p14726, p14729, p14731, p14728, and p13567 (used for complementation analyses of mutants Δ*DR0007*Ω*hph*, Δ*DR0008*Ω*hph*, Δ*DR0007*ΔD*R0008*Ω*hph* and Δ*DR0009*Ω*hph* respectively), p13564 (for complementation analyses of mutant Δ*DR0265*Ω*hph*) and p13563 (for complementation analyses of mutant Δ*DR2462*Ω*hph*).

### Mapping Tn*5* insertions sites into *D*. *radiodurans* genome

Insertion mutants were mapped by the arbitrary-primed (AP)-PCR procedure [91,92], using GoTaq Flexi DNA polymerase (Promega). The first PCR round was performed in a final volume of 50μL with 1 μL genomic DNA from single Hyg^R^ colonies as template. The arbitrary primer (ARB1c) was paired either with a primer specific for the 5’ end of the mini-Tn*5* (Tn5-212) or with a primer specific for the 3’ end of the mini-Tn*5* (Tn5-991), both at a final concentration of 0.8 μM and PCR was performed as follows: 2 min 95°C, 6 cycles of 45 s 95°C, 45 s 30°C, 1 min 30 s 72°C; 30 cycles of 45 s 95°C, 45 s 45°C, 2 min 72°C; and finally 72°C for 5 min. The second round was performed in a final volume of 50 μL with 5 μL of the purified PCR product from round 1 as template. A second arbitrary primer (ARB3) was paired either with the Tn5-166 primer (5’ end of Tn*5*) or with the Tn5-1055 primer (3’ end of Tn*5*), each at a final concentration of 0.8 μM and PCR was performed as follows: 2 min 95°C, 30 cycles (45 s 95°C, 45 s 52°C, 2 min 72°C); 72°C for 5 min. The products of this PCR were directly sequenced with the SeqRE primer (5’ end of Tn*5*) or with the EB89 primer (3’ end of Tn*5*) by Cogenics (Meylan, France). Oligonucleotides used are listed in S3 Table.

### Procedure for *in vivo* transposition of mini-Tn*5*-*hph* into *D*. *radiodurans*

We have used the *D*. *radiodurans* GY10973 strain expressing a chromosomal copy of *lacI* under the control of the P_*tufA*_ promoter as a recipient, to ensure a non-toxic level of the transposase during the mutagenesis process. The isolation of Tn*5* insertion mutants was performed in two steps as follows. First, the *D*. *radiodurans* GY10973 strain was transformed with the Tn*5*-*hph* delivery plasmid p13554 and transformants were selected at permissive temperature (30°C) on TGY agar supplemented with both hygromycin and spectinomycin. Ten independent Hyg^R^ Spc^R^ colonies were then pooled and resuspended into 200 μl of TGY2X. 15 μl of this resuspension was inoculated into 3 ml TGY2X supplemented with spectinomycin and incubated at 30°C with shaking to an A_650_ ~ 0.1 (transposition step). This culture was finally serially diluted and plated onto both TGY agar without antibiotic to quantitate the viable cells and onto TGY agar supplemented with hygromycin and incubated at a non-permissive temperature (37°C) to simultaneously select for Tn*5*-*hph* insertion mutants and cure the delivery plasmid.

### Calculation of the number of predicted inactivated ORFs in the Tn*5* insertion mutant library

Given that Tn*5* insertion is random, the number of independent single-gene insertions in *D*. *radiodurans* required to inactivate 99% of the genes can be calculated using the following formula [43]: P = 1 – (1 - [x/g])^n^, where P = probability of finding one transposon insertion within a given gene (0.99), x = average length of a *Deinococcal* gene (1011 bp), g = *D*. *radiodurans* genome size (3284156 bp), and n = number of independent insertion mutants. The number of genes inactivated among the 6207 insertion mutants analysed in our screening was estimated, based on Poisson’s law: P (k) = (λ^k^/k!)e^-λ^, where k = number of insertions within a given gene and λ = the average number of insertions per gene. Given that the *D*. *radiodurans* genome encodes 3,195 predicted protein-encoding genes [73], one can estimate λ = 6,207/3,195 = 1.9427 insertions per gene. Therefore, the probability of having no insertions within a given gene is P(0) = (λ^0^/0!)e^-λ^ = e^-λ^ = 0.1433, indicating that approximately 14% of the genes were not inactivated among the 6207 mutants.

### Library screening for sensitivity to DNA damaging agents (γ, UV-irradiation and MMC treatment)

First, the Hyg^R^ Spc^S^ insertion mutants selected at non-permissive temperature (37°C) were homogenotized (for non-essential genes) as follows: the colonies were arrayed to 96-well microtiter plates containing 100 μL of TGY2X broth supplemented with hygromycin (50 μg/mL) per well, grown statically at 37°C for one day, followed by three serial replica always on hygromycin supplemented TGY agar plates. The purified individual mutant clones (homogenotized or partially homogenotized) were replica plated on TGY-agar plates containing hygromycin and exposed either to either γ-rays (at a dose of 7500 Gy) or UV-rays (at a dose of 600 J m^-2^) or onto TGY plates supplemented with mitomycin C (at 30 ng/mL). To confirm their radiosensitive phenotype, the candidates arising from the first screening were then grown overnight in TGY2X supplemented with hygromycin and 5 μl of undiluted or 1/10^th^ diluted culture were spotted on plates subsequently γ-irradiated (7500 Gy) or UV-irradiated (600 J m^-2^) or onto plates supplemented with MMC. To validate our screening and homogenotization procedure, we have included in our screen a control mutant by transformation of the tester strain GY10973 with *in vitro* engineered *pprA* gene inactivated by insertion of the same hygromycin cassette. Lastly, to ensure that the observed radiosensitive phenotypes are genetically linked to Tn*5* insertion, all candidates sensitive to DNA damaging agents isolated in the GY10973 strain were further confirmed by backcross to the wild type *D*. *radiodurans* R1 strain (see Fig 1B for screening procedure).

### Screening of *D*. *radiodurans* mutants for sensitivity to H_2_O_2_ stress

All mutants sensitive to γ-rays and further confirmed by backcross were analysed for their sensitivity to hydrogen peroxide (Sigma-Aldrich) by using the disc inhibition assay as follows. 1 mL of exponential phase cultures (A_650_ ~ 0.2) was spread-plated onto TGY plates, and sterilized 6-mm-diameter filter paper discs (Dominique Dutscher) were placed on the agar surface. Then, 10 μL of various concentrations (50, 100, 120, 150 and 200 mM) of H_2_O_2_ (freshly diluted in 10 mM potassium phosphate buffer) was spotted onto each disc. After incubation at 30°C for 2 days, the diameters of the growth inhibition zones were measured. The mutants were classified into four categories depending on the diameter of growth inhibition area as described in Fig 2.

### Survival of *D*. *radiodurans* to different DNA damaging agents

For all experiments, the colonies were counted after 3–4 days incubation at 30°C.

- **Gamma irradiation**. Bacteria were grown in TGY2X or in TGY2X media supplemented with spectinomycin when they contained a plasmid or with 10 mM IPTG and spectinomycin when they contained plasmids p11559 or derivatives to an A_650_ ≈ 1. The cultures were concentrated six times in TGY2X and irradiated on ice with a ^137^Cs irradiation system (Institut Curie, Orsay, France). Following irradiation, diluted samples were plated on TGY plates, or TGY plates supplemented with spectinomycin when bacteria contained plasmid p11559 or derivatives and incubated at 30°C before the colonies were counted.
- **UV irradiation**. Bacterial cultures at an A_650_ = 1 were serially diluted in TGY and plated on TGY agar plates (or TGY plates supplemented with spectinomycin when bacteria contained plasmid p11559 or derivatives), subsequently exposed to UV light at a dose rate of 3.5 J/m^2^/s and incubated at 30°C.
- **MMC treatment**. Bacterial cultures at an A_650_ = 1 were serially diluted in TGY and plated on TGY plates (or TGY plates supplemented with spectinomycin when bacteria contained plasmid p11559 or derivatives) supplemented with MMC at final concentrations of 40 and 60 ng mL^-1^ and incubated at 30°C.

### Kinetics of DNA repair measured by pulse-field gel electrophoresis

Non-irradiated or irradiated (3,800 Gy) cultures were diluted in TGY2X to an A_650_ = 0.3 and incubated at 30°C. At different post-irradiation recovery times, samples (5 ml) were harvested to prepare DNA plugs as described [93]. The agarose embedded DNA plugs were digested for 16 h at 37°C with 60 units of *Not*I restriction enzyme. After digestion, the plugs were subjected to pulsed field gel electrophoresis for 28 hours at 10°C using a CHEF MAPPER electrophoresis system (Biorad) with the following conditions: 5.5 V / cm, linear pulse of 40 s, and a switching angle of 120° (- 60° to + 60°).

### Fluorescence microscopy

The cells were fixed by adding toluene (3% final concentration) to culture aliquots, and kept at 4°C. DNA and cell membranes were stained with DAPI (40 μg/ml) and FM4-64 (50μg/ml), respectively, as previously described [24] and spotted on a thin layer of TGY2X agarose 1% for microscopy observation. The stained cells were observed using a Leica DMIRE2 microscope with a 100X objective and the appropriate fluorescence filters.

## Supporting Information

S1 Fig Functional categories of Tn*5*-inserted genes in mutants sensitive to DNA damaging agents isolated by screening onto TGY agar plates. (PDF)

S1 Table Master screening data of backross Tn*5* insertion mutants for sensitivity to γ- and UV rays, MMC or hydrogen peroxide (H2O2). (PDF)

S2 Table Bacterial strains and plasmids and corresponding references. (PDF)

S3 Table Overview of primers used for strains construction, cloning, mutagenesis, and AP-PCR experiments. (PDF)
