# Supplementary material for: Identification of New Genes Contributing to the Extreme Radioresistance of Deinococcus radiodurans Using a Tn5-Based Transposon Mutant Library
Source: PLoS One. 2015 Apr 17;10(4):e0124358. doi: 10.1371/journal.pone.0124358 (PMC4401554; doi:10.1371/journal.pone.0124358)

Figure S1. Functional categories of Tn*5*-inserted genes in mutants sensitive to DNA damaging agents isolated by screening onto TGY agar plates (see Materials and Methods and Fig. 1B for details of library screening procedure and S1 Table for detailed description of the 208 mutants obtained). Coloring is according to COG.

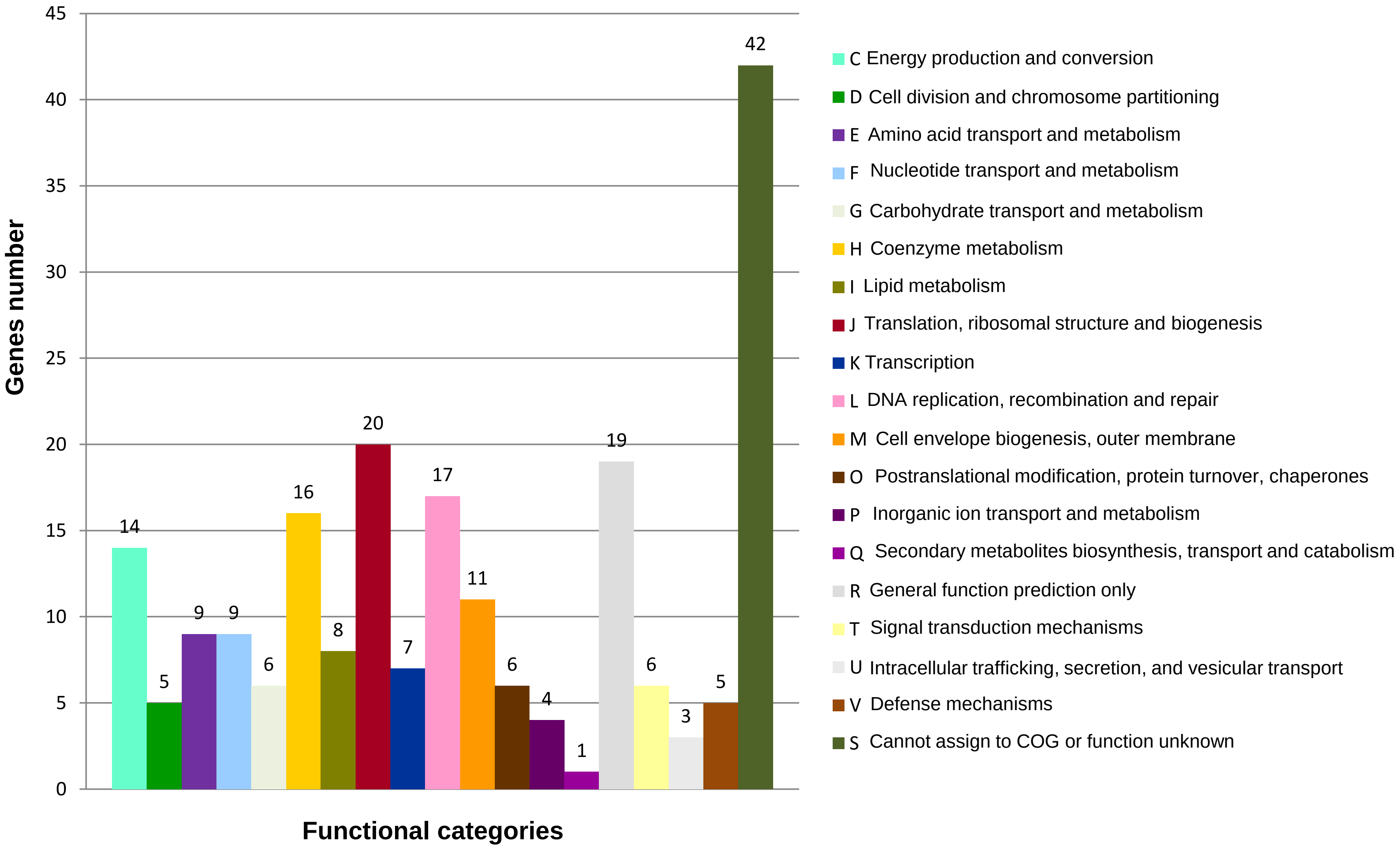

Supplement: S1 Fig — (PDF) [file pone.0124358.s001.pdf]
