# Supplementary material for: Identification of New Genes Contributing to the Extreme Radioresistance of Deinococcus radiodurans Using a Tn5-Based Transposon Mutant Library
Source: PLoS One. 2015 Apr 17;10(4):e0124358. doi: 10.1371/journal.pone.0124358 (PMC4401554; doi:10.1371/journal.pone.0124358)
Supplement: S1 Table — (PDF) [file pone.0124358.s002.pdf]

Table S1. Master screening data of backross Tn*5* insertion mutants for sensitivity to γ-and UV rays, Mitomycin C (MMC) or hydrogen peroxide (H_2O_2)

[a]identity of disrupted sequences was based on the genomic sequence data and updated annotation (2004), Koonin's group (http://www.usuhs.mil/pat/deinococcus/dradioduransgenome.html)

[b]onto TGY1X agar plates at the dose or concentration indicated; SS, highly sensitive; S, sensitive ; s, slightly sensitive (see Table 1)

[c]H2O2 disc assay as described in Materials and Methods and Figure 2

[d]in TGY2X liquid culture as described in Materials and Methods

[e]determined by diagnostic PCR, H: homozygous insertion mutant, h: heterozygous insertion mutant

[o]gene is located in a putative operon (predicted by FGENESB (www.softberry.com).

[p]transposon inserted in the putative promoter region (predicted by BPROM (www.softberry.com)

[u]transposon inserted in the upstream region of the corresponding CDS.

Strain (backross)	Inactivated locus	[a]COG-name, putative function	γ sensitivity[b]	MMC sensitivity[b]	UV sensitivity[b]	H_2O_2 sensitivity[c]	[d]Survival to γ (%)		[e]Homozygous/ heterozygous	Comments
dose or concentration			7.5 kGy	40 ngmL^{-1}	600 J m^{-2}		10 kGy	15 kGy		
wild type control ATCC 13939							**85**	**38**		
GY14341	DR0001	DNA Polymerase III, β-subunit, DnaN	s	s	R		**86,5**	**33,4**	h	essential gene in bacteria
GY14838	DR0007	Uncharacterized conserved protein	S	SS	s	R	**10,1**	**0,5**	H	
GY14887	DR0038	Serine hydroxymethyltransferase GlyA	s	s	s	R	**41**	**2,1**	H	2 different insertions in this ORF
GY15464	[(p)]DR0084	GTP1/OBG family GTPase	s	R	R	R	**19,8**	**11,8**	h	essential gene in bacteria
GY14344	DR0093	Phytoene dehydrogenase, crtO	s	R	R	R	**70,2**	**18,5**	H	Probably participates in pigment biosynthesis
GY14343	DR0098	30S Ribosomal protein S6, RpsF	s	R	R		**45**	**29,4**	h	
GY15448	DR0108	Conserved membrane protein, transporter	S	S	S	R	**72,4**	**37,9**	H	
GY14946	DR0118	Acetyl-CoA carboxylase, Biotin carboxyl carrier protein, AccB	S	R	R	s	**100**	**28**	h	
GY15411	DR0126	Chaperone protein DnaJ (*dnaJ-1*)	S	R	s	R	**74,8**	**55,4**	H	3 different insertions in this ORF
GY15357	DR0137	Predicted type IV restriction endonuclease	s	R	R		**69**	**11,3**	H	
GY14953	DR0138	Membrane protein, similar to slr0686 of Synechocystis	s	R	R		**91,4**	**29,1**	H	

GY15354	DR0145	Probable cell surface protein	S	R	R	R	**49,4**	**9,78**	H	
GY14925	DR0148	Valyl-tRNA synthetase, valS	s	R	R		**88,4**	**29,1**	h	essential gene in bacteria
GY14324	DR0156	Riboflavin synthase beta subunit, RisB	S	R	R	R	**57,9**	**19,5**	H	
GY15445	DR0167	DNA repair regulator protein, IrrE	S	SS	SS	S	**1**	**0,01**	H	2 different insertions in this ORF
GY15211	DR0198	Recombinational DNA repair protein, RecR	SS	S	s	s	**23,1**	**13,52**	h	
GY15466	DR0215	NIfS family of aminotransferase	SS	R	S	R	**29,7**	**16**	H	
GY15279	DR0243	Arginine decarboxylase (spermidine biosynthesis)	S	R	R	R	**61**	**23,5**	H	
GY15428	DR0247	Phosphopantetheinyl transferase, AcpS	SS	R	R	R	**66,38**	**25,85**	h	
GY15362	between DR0251 and DR0252	intergenic region	S	R	R	R	**24,2**	**0,1**	H	
GY14859	DR0256	Dihydrolipoamide acyltransferase, (E2) component, Odp2	SS	S	SS	R	**78,8**	**33,3**	H	
GY15422	DR0257	Pyruvate dehydrogenase complex, dehydrogenase (E1) component, Odp1	S	SS	SS	R	**77,1**	**33,8**	H	
GY14335	DR0265	HTH transcriptional regulator, GntR family	S	R	S	S	**19,3**	**0,6**	H	
GY15333	DR0266	molybdenum cofactor biosynthesis protein A, MoaA	S	R	s	R	**20,1**	**8,1**	H	
GY14840	DR0287	2-oxoglutarate dehydrogenase complex, dehydrogenase (E1) component, Odo1	S	SS	SS	R	**100**	**48,7**	H	2 different insertions in this ORF
GY14351	DR0295	Protein phosphatase, calcineurin like phosphoesterase	S	R	R	R	**96,8**	**43,9**	H	
GY14393	DR0297	UDP-N-acetylmuramyl tripeptide synthetase	s	R	s		**62,7**	**10,7**	h	essential gene in bacteria
GY15366	DR0315	30S ribosomal protein S19, RpsS	S	R	s	s	**39,2**	**2,71**	h	essential gene
GY14933	DR0342	cytochrome complex iron-sulfur subunit (Rieske like Fe-S protein)	S	s	s	R	**51,2**	**4,61**	H	

GY15463	[u]DR0347	Cytochrome C-Type biogenesis protein CycJ	S	R	R	R	**4,5**	**1,2**		
GY15269	DR0348	cytochrome c-type biogenesis heme exporter protein C, cycZ	SS	R	R	s	**43,5**	**13,9**	h	
GY15278	DR0359	Predicted membrane protein	S	R	R	R	**81,9**	**34,6**	H	
GY15320	DR0362	D-alanyl-alanine synthetase A, Ddl	S	R	R	R	**42,9**	**19,5**	h	
GY15423	DR0372	Lysyl-tRNA synthetase, LysS	SS	R	R	s	**62,1**	**44,8**	h	essential gene
GY15267	DR0379	Outer membrane protein	s	S	s		**65,6**	**41,9**	h	2 different insertions in this ORF
GY14852	DR0383	S-layer-like array-like protein	SS	R	R	R	**78,4**	**33,2**	H	
GY15259	DR0386	1-hydroxy-2-methyl-2-(e)-butenyl-4-diphosphate synthase, GcpE ortholog	SS	R	R	s	**54,8**	**25,3**	h	
GY14849	DR0400	DNA segregation ATPase, FtsK	R	SS	R		ND		h	essential gene
GY14333	DR0415	Esterase of the acetylcholinesterase family	S	R	R	R	**65,5**	**16,3**	H	
GY15286	DR0430	RtcB family protein	s	R	R		**46,5**	**11,5**		
GY14328	DR0433	Beta-lactamase class A	S	s	R	R	**66,5**	**20,3**	h	
GY15283	DR0439	HesB family of small cysteine rich proteins	S	R	s	R	**71**	**16,1**	H	
GY15209	DR0454	BacA bacitracin resistance protein	S	s	R	R	**75,8**	**29,5**	h	
GY14833	DR0487	ABC-type transport system, involved in lipoprotein release, permease component	S	R	R	R	**72,7**	**11,7**	H	
GY14936	DR0507	DNA polymerase III subunit alpha, DnaE	s	R	R		**56**	**24**	h	
GY15309	DR0511	Nitrate transport ATPase, NrtC	S	R	R	R	**60,6**	**18,3**	H	
GY15348	DR0555	Glutamate-1-semialdehyde aminotransferase	SS	s	s	R	**43**	**7,8**	h	
GY14841	DR0573	Secreted surface protein, uncharacterized	SS	S	S	R	**100**	**24,4**	H	5 different insertions in this ORF

GY14858	DR0577	Membrane bound sensor histidine kinase	SS	s	R	s	**72**	**38**	h	2 different insertions in this ORF
GY15246	DR0579	DNA/pantothenate metabolism flavoprotein, Dfp	SS	s	R	s	**76**	**29,7**	h	2 different insertions in this ORF
GY15210	DR0584	Formyltetrahydrofolate hydrolase, PurU	SS	s	R	s	**35,8**	**7,4**	H	
GY15465	DR0638	Uncharacterized conserved membrane protein	SS	R	R	R	**17,3**	**6,4**	h	
GY14930	DR0679	Small nucleotidyltransferase-like protein	S	s	s	S	**28,3**	**1,4**	H	
GY15416	DR0744	Signal transduction histidine kinase	S	S	SS	R	**60,5**	**36,1**	h	
GY14999	DR0751	Septum formation topological specificity factor, MinE	S	R	s	R	**100**	**14,8**	h	
GY14391	(p)DR0755	Ribosomal protein L19	SS	s	R	s	**100**	**58,8**	h	essential gene
GY15404	DR0764	Lipoate-protein ligase, LipB	SS	S	S	R	**76,36**	**30,93**	h	2 different insertions in this ORF
GY14325	DR0777	3-dehydroquinate synthase, AroB	S	s	R	R	**100**	**34,8**	h	very slow growth
GY15230	DR0824	Similar to N-ethylammeline chlorohydrolase, TIM barrel metal-dependent hydrolase	S	R	s	R	**85**	**24,96**	h	
GY14326	DR0826	DHH superfamily hydrolase	SS	SS	s	s	**33,1**	**2,5**	H	
GY15247	DR0845	Ribose 5-phosphate isomerase, RpiA	S	S	R	s	**70,5**	**22**	H	
GY14914	DR0906	DNA gyrase B subunit, GyrB	S	R	R	s	**91,5**	**37,3**	h	essential gene
GY14337	DR0913	Uncharacterized conserved protein	SS	s	R	R	**78,8**	**23,9**	h	
GY14331	DR0914	16S RNA methylase RsmC	S	S	R	R	**49,7**	**8,5**	H	2 different insertions in this ORF
GY15263	DR0975	ADP-ribose pyrophosphatase (MutT/NUDIX family hydrolase)	SS	S	R	R	**84**	**12,1**	H	
GY15407	DR0983	tRNA nucleotidyltransferase, Cca	SS	s	S	s	**38,5**	**22,5**	h	
GY15425	DR0997	ddrI, CRP/FNR family transcriptional regulator	SS	SS	SS	s	**4,7**	**0,4**	h	
GY15338	DR1008	Riboflavin kinase, RibF	SS	?	?	R	**70,2**	**48,2**	h	

GY14390	DR1050	Predicted protein	s	R	R		**53,6**	**26,8**	H	
GY15249	DR1062	FemA-like protein	S	R	R	s	**63,1**	**29,7**	h	
GY15203	DR1086	Alanine racemase, Alr	S	R	R	s	**52**	**18,6**	h	
GY15254	DR1096	3-phosphoshikimate 1-carboxyvinyltransferase	S	R	R	R	**54,2**	**25**	h	
GY15325	DR1125	Predicted membrane protein	S	R	R		**83,9**	**25,6**	h	
GY14836	**(o)DR1130**	Protoporphyrinogen IX and coproporphyrinogen III oxidase HemY	SS	s	R	R	**62,3**	**10,9**	h	
GY14831	**(o)DR1131**	Protoheme ferro-lyase (ferrochelatase), HemZ	S	R	R	S	**33,3**	**8,9**	h	
GY14830	**(o)DR1133**	Uroporphyrinogen-III decarboxylase	S	R	R	s	**54**	**6,3**	h	
GY15322	DR1140	Uncharacterized conserved protein, probably lipoprotein	SS	SS	SS	a refaire	**60,5**	**31,9**	H	
GY14362	DR1152	Releated to double stranded beta-helix domain of AraC, YlbA E.coli ortholog	s	R	R		**38,7**	**7,1**	h	2 different insertions in this ORF
GY14354	DR1164	Pantothenate synthetase, PanC	SS	S	S	R	**51,6**	**4,1**	H	
GY14360	DR1167	DedA-like membrane protein	S	R	R	R	**74**	**14,1**	H	
GY15308	DR1203	N-terminal domain of LmbE family protein, contains HPDD motif	SS	R	R	R	**79,6**	**44,2**	H	
GY15213	DR1207	Fusion cell cycle protein MesJ and cytosine deaminase	S	S	s	S	**30,7**	**10**	h	
GY15285	DR1217	HTH transcriptional regulator	SS	R	R	R	**93,8**	**49,3**	H	
GY14379	DR1222	Acetyl-xylan esterase, NodB-like deacetylase	s	R	s		**45,3**	**13**		
GY15462	DR1244	DNA polymerase III, delta subunit, holA	s	s	s		**65,1**	**31,3**	h	essential gene
GY15355	DR1268	DinB/YfiT family of proteins	s	R	R		**47,3**	**8,3**	h	
GY15418	DR1274	Holliday junction helicase, DNA-binding subunit, RuvA	S	SS	SS	s	**18,81**	**5,42**	h	
GY15310	DR1311	Methionine peptidase, Map	S	R	R	R	**76,2**	**26,6**	h	

GY14381	DR1321	Signal peptidase I	S	S	S	R	**42,6**	**4,56**	H	
GY15234	DR1335	Isoleucyl-tRNA synthetase, IleS	SS	R	R	s	**42,1**	**27,4**	h	essential gene
GY14967	DR1354	Nuclease subunit of the excinuclease complex,UvrC	s	S	s	R	**5,03**	**0,03**	H	2 different insertions in this ORF
GY15352	DR1374	DNA topoisomerase I	SS	s	s	R	**12,8**	**1,88**	h	essential gene; 2 different insertions in this ORF
GY15225	DR1392	Conserved hypothetical protein	SS	S	S	R	**62,9**	**17,8**	h	
GY15328	DR1395	Geranylgeranyl pyrophosphate synthase, IspA	SS	R	R	s	**62,4**	**41,6**	h	2 different insertions in this ORF
GY15274	DR1402	HTH transcriptional regulator, TetR/ArcR family	S	R	R	s	**90,2**	**18,1**	h	
GY15227	DR1415	Orthinine/acetylornithine aminotransferase, ArgD	S	R	s	R	**87,2**	**33,2**	h	
GY15461	DR1446	Uncharacterized conserved protein	s		R		**65,2**	**38,6**	H	
GY15000	DR1456	Phosphoribosylpyrophosphate synthetase	SS	s	S	s	**58,3**	**25,8**	h	2 different insertions in this ORF
GY15268	DR1460	Predicted extracellular protein	s	s	s		**44,1**	**9,12**	H	
GY14857	DR1461	Predicted extracellular protein	s	S	SS		**85,9**	**47,9**	H	2 different insertions in this ORF; very slow growth
GY14972	DR1469	N-acetylmuramoyl-L-alanine amidase	s	R	R		**45,7**	**17,2**	h	
GY15248	(p)DR1475	1-deoxy-D-xylulose-5-phosphate synthase, transketolase, Dxs	SS	S	S	R	**57,49**	**9,5**	H	second insertion in this ORF
GY14374	DR1477	DNA repair protein, RecN	S	SS	S	R	**28,93**	**5,5**	H	2 different insertions in this ORF
GY15431	DR1481	Chlorite dismutase family enzyme	SS	R	SS	s	**64,59**	**28,99**	h	
GY15294	DR1488	HTH transcriptional regulator, LytR ortholog	s	S	s		**81,31**	**22,77**	H	
GY14835	DR1507	Membrane-associated Zn-dependent protease	S	R	R	R	**100**	**23,1**	H	
GY14327	DR1514	2-phosphoglycerate kinase	S	S	SS	R	**97,8**	**8,6**	h	

GY15228	DR1525	Fructokinase RbsK ortholog	SS	SS	S	s	**48,7**	**3,43**	H	additional insertion in the putative promoter region
GY15252	DR1574	Phosphatidylserine decarboxylase	S	R	R		**57,8**	**33,5**	H	
GY14843	DR1607	Uncharacterized conserved protein	S	s	R	R	**55,2**	**7,88**	h	very slow growth
GY15358	DR1612	Roadblock/LC7 domain	s	R	R		**78,8**	**11,5**		
GY15353	DR1709	NRAMP family membrane transporter	s	R	s	R	**28,8**	**4,6**	h	
GY15356	DR1740	Protein containing N-terminal CDNR domain	s	R	R		**38,5**	**5,5**	h	
GY14396	DR1771	Excinuclease ATPase subunit, UvrA-1	S	SS	s	s	**5,7**	**0,3**	h	3 different insertions in this ORF
GY14377	DR1774	Predicted protein	s	R	s		**62,9**	**11,3**	H	
GY15350	DR1794	ApbE family protein	s	R	R		**42,4**	**5,6**	h	
GY15232	DR1797	Transcription termination-antitermination factor NusA	SS	R	R	s	**85,9**	**36,5**	h	
GY14378	DR1799	Translation initiation factor 2 (IF-2; GTPase)	S	R	R	s	**82,1**	**18,9**	h	essential gene
GY15256	DR1833	Predicted protein	S	R	R	R	**69,8**	**19,1**	H	
GY14853	DR1854	ComEC/Rec2 ortholog	SS	R	R	R	**83,3**	**29,1**	H	2 different insertions in this ORF
GY14394	DR1869	Polyferredoxin	S	s	s	R	**52**	**43,3**	h	
GY15250	DR1887	Membrane protein	SS	R	R	s	**78,2**	**45,4**	h	
GY15270	DR1915	Hydrolase of the alpha/beta superfamily	S	R	R	R	**52,7**	**14,9**	H	
GY15206	DR1916	RecG superfamily II helicase	s	SS	S	R	**23,05**	**4,1**	H	2 different insertions in this ORF
GY15296	(u)DR1940	Heat shock protein HSLJ, three homologous domain fused	SS	R	R	R	**76,8**	**25,6**	H	
GY15237	DR1945	(acyl-carrier-protein) S-malonyltransferase, FabD	SS	s	s	s	**67**	**47,5**	h	
GY14339	DR1950	Aromatic compound dioxygenase, ferredoxin	s	S	R	R	**24,5**	**0,6**	h	

GY14332	DR1983	Ribosomal protein S1, RpsA	S	R	R	s	**70,7**	**48,5**	h	essential gene
GY14338	DR1991	tRNA pseudouridine synthase D, TruD	SS	R	R	R	**100**	**69,2**	H	
GY14986	DR1998	Catalase, KatA	SS	R	R	SS	**48**	**15,5**	H	2 different insertions in this ORF
GY15245	(u)DR2024	Uncharacterized conserved membrane protein	S	R	R	R	**64,5**	**44,6**	H	insertion 5 nt upstream the initiation codon
GY14850	DR2058	Uncharacterized secreted protein	S	R	R	R	**51,9**	**4,58**	H	
GY15291	DR2067	Transcription termination factor NusB	SS	S	SS	R	**99,6**	**80,63**	H	
GY14398	DR2069	NAD-dependent DNA ligase, DnlJ	S	s	s		**95,7**	**20,3**	h	essential gene; 3 different insertions in this ORF
GY15436	DR2081	Threonyl-tRNA synthetase	S	R	s	s	**67,9**	**46,2**	h	essential gene
GY15349	DR2106	Cysteine desulfurase activator SufB	SS	s	s	R	**43,2**	**7,57**	h	2 different insertions in this ORF
GY15243	DR2114	Ribosomal protein L30, RpmD	SS	R	R	s	**51,7**	**65,9**	h	essential gene
GY15329	DR2116	Preprotein translocase SecY	SS	R	R	s	**78,9**	**23**	h	
GY15251	DR2149	Conserved membrane protein	SS	R	R	s	**71**	**43,6**	h	
GY14943	DR2151	RnpA Ribonuclease P protein component	S	R	R	R	**45,4**	**0,3**	H	
GY15297	DR2156	Uncharacterized conserved protein	SS	R	R	s	**60,7**	**7,1**	H	2 different insertions in this ORF
GY14847	DR2166	Purine-nucleoside phosphorylase, DeoD	s	s	R		**55,7**	**8,8**	H	
GY15433	(u)DR2188	Leucyl aminopeptidase (aminopeptidase T)	SS	R	s	s	**63,2**	**16,4**	h	
GY14834	DR2195/DR 2196	intergenic region	S	R	R	R	**70,7**	**63,1**	H	
GY14350	DR2200	Orotidine-5'-phosphate decarboxylase	s	s	R		**75,6**	**30,4**	h	
GY15212	(u)DR2210	Protein with conserved cysteines similar to UmoC of Proteus mirabilis	s	s	s		**52,5**	**15**	H	
GY14862	DR2213	Carbamoyl phosphate synthetase homolog	SS	R	R	R	**61,7**	**30,5**	H	

GY14966	DR2256	Transketolase, Tkt	s	s	s		**52,4**	**30,8**	h	
GY15327	DR2275	Helicase subunit of the DNA excision repair complex, UvrB	S	SS	R	R	**6,2**	**0,7**	H	2 different insertions in this ORF
GY14353	DR2289	Guanylate kinase	S	s	R	R	**62,3**	**20,9**	h	
GY15363	DR2300	Alanyl tRNA synthetase, AlaS	S	s	s	s	**23,2**	**2,69**	h	
GY14330	DR2320	Uncharacterized conserved protein, related to organic solvent tolerance protein OstA	S	S	S	R	**99,8**	**90**	H	2 different insertions in this ORF; very slow growth
GY15216	DR2341	BirA bifuctional protein, biotin operon repressor, HTH and biotin-protein ligase	s	s	S		**74,5**	**6,4**	H	
GY15444	DR2357	Phenylalanyl-tRNA synthetase beta chain	SS	R	s	R	**63**	**30,6**	h	essential gene
GY14364	DR2370	Pyruvate dehydrogenase complex, dihydrolipoamide dehydrogenase (E3) component	S	s	s	R	**78,5**	**26,3**	h	
GY14861	DR2374	Ribonucleotide reductase, archeal type	S	s	R	S	**47,9**	**23**	h	essential gene
GY14329	DR2417m	YkqC ortholog, hydrolase of the metallo-beta-lactamase superfamily	S	s	R	S	**66**	**12,2**	h	
GY14323	DR2418	Response regulator, DrRRA	**S**	**SS**	R	R	**32**	**1,51**	H	2 different insertions in this ORF
GY14860	DR2430	Conserved membrane protein, probably permease	S	s	s	R	**53,7**	**18,5**	H	
GY15262	DR2431	Conserved membrane protein, probably permease	S	S	s	R	**71,2**	**17**	h	2 different insertions in this ORF
GY15437	DR2434	Peptide deformylase	S	R	s	s	**53,1**	**23,4**	h	
GY15202	DR2435	Methionyl-tRNA formyltransferase	SS	R	R	s	**69,3**	**45,6**	h	essential gene
GY14837	DR2462	Rnase Y superfamily	SS	S	SS	R	**30,1**	**0,9**	H	2 different insertions in this ORF
GY15447	DR2497	Cell division protein, FtsW/RodA/SpoVE family	S	R	S	R	**60,8**	**38,5**	h	essential gene
GY14361	DR2508	Hexagonally packed intermediate-layer (HPI) surface protein	s	R	R		**56,7**	**8,5**	H	
GY14829	DR2511	Signal peptidase II-like protein, Bacillus YaaT ortholog	S	R	s	R	**60,1**	**8,8**	H	
GY14988	DR2514	O-acyltransferase PlsC/PlsB family	s	s	s		**65**	**25,1**	h	

GY15298	DR2518	Pkn2 type serine threonine kinase	SS	SS	SS	s	**26,3**	**6**	H	
GY15219	DR2552	Conserved protein, related to aminodeoxychorismate lyase, YceG ortholog	S	SS	SS	R	**29,4**	**11,5**	H	slow growth
GY15261	DR2557	Beta-lactamase superfamily of Zn-dependent hydrolases	s	R	R		**47,3**	**6,7**	H	
GY15229	DR2572	Predicted membrane protein	s	R	s	R	**38,9**	**3,4**		2 different insertions in this ORF
GY15426	DR2576	DHH family phosphohydrolase	SS	R	R	s	**72,8**	**35,2**	h	
GY15427	DR2577	S-layer protein	S	SS	SS	R	**100**	**96,9**	H	2 different insertions in this ORF
GY14848	DR2586	Exonuclease VII small subunit XseB	S	R	R	R	**42,9**	**28,8**	H	
GY15217	DR2602	Acetate/propionate kinase	SS	s	R	R	**87,8**	**31,3**	h	
GY14399	DR2606	Primosomal protein N' (replication factor Y) - superfamily II helicase	S	SS	S	R	**85,4**	**4,22**	H	
GY15224	DR2611	Glutaminyl tRNA synthetase fused to pet112, class I	SS	R	s	s	**73,8**	**8,57**	h	2 different insertions in this ORF
GY15351	DR2613/DR 2614	intergenic region	s	R	R	R	**33,2**	**5,26**	H	
GY15207	DR2615	Ketopantoate hydroxymethyltransferase	SS	SS	S	R	**44,5**	**4,28**	H	2 different insertions in this ORF
GY15215	DR2617	Cytochrome AA3 controlling protein, CtaA	SS	s	s	R	**100**	**27,4**	h	2 insertions in this ORF
GY14340	DR2619	Cytochrome c oxidase, subunit II, CtaC, authentic frameshift	+/-	+/-	R		**40,5**	**23**	h	
GY14902	DR2620	Cytochrome oxidase subunit I, Cox1	+/-	R	R		**39,1**	**5,32**	h	
GY14320	DR2630	Thymidylate synthase	SS	SS	S	s	**33,5**	**10,3**	H	2 insertions in this ORF
Chromosome II										
GY15253	DRA0011	Uroporphyrinogen-III methylase and uroporphyrinogen-III synthase	SS	S	S	R	**68,1**	**33,1**	H	
GY14336	DRA0013	Sulfite reductase, beta subunit NirA	S	R	R	R	**84,6**	**29,1**	H	

GY15258	DRA0022	Uncharacterized conserved secreted enzyme	s	R	R	R	**40,5**	**4,83**	h	
GY15289	DRA0033	Related to exopolysaccharide export protein, EpsB, MinD/ParA family ATPase	s	R	R		**55,1**	**10,5**	H	
GY14998	DRA0034	Glycosyltransferase involved in lipopolysaccharide synthesis, RfbP	S	s	s	R	**92,3**	**14**	H	
GY15271	DRA0041	dTDP-D-glucose 4,6-dehydratase, RfbB	S	R	R	R	**50,7**	**14,5**	H	
GY15459	DRA0044	dTDP-4-dehydrorhamnose reductase, RfbD/C	s				**33,9**	**19**	h	
GY14845	DRA0065	DNA-binding protein HU, HupA	S	R	R				h	essential gene
GY14828	DRA0132	N-acetylglucosaminyl-phosphatidylinositol deacetylase related protein	s	R	R		**73,8**	**29,7**	H	
GY15440	DRA0229	Predicted protein	S	R	R	R	**60,9**	**34,5**	h	
GY15260	DRA0244	Oxygen-independent coproporphyrinogen III oxidase	s	R	R		**60,8**	**25**	H	
GY15239	DRA0268	Predicted protein	s	R	R		**60,7**	**27,3**	H	
GY14895	DRA0274	Flavin-containing amine oxidase	SS	s	R	R	**84,4**	**20,8**	h	2 different insertions in this ORF
GY14975	DRA0276	Malate dehydrogenase	s	R	R	R	**39,4**	**4,2**		
GY14854	DRA0292	Predicted protein, probably secreted	s	R	R		**72**	**21,8**	h	
GY15280	DRA0297	Sensor regulator, TPR repeats domain (N-terminal) and GGEEF domain	s	R	R		**62,6**	**34,3**	H	
GY14385	DRA0338	L-kynurenine hydrolase	S	R	R	R	**62,3**	**24**	H	
GY14881	DRA0346	PprA protein, involved in DNA damage resistance mechanisms	SS	SS	SS	R	**0,4**	**0,1**	H	2 different insertions in this ORF
Megaplasmid										
GY15439	DRB0075	PTS system, multiphosphoryl transfer protein, domains EI, HPr, and fructose specific EIIA	S	S	s	R	**74,4**	**44,4**	H	
GY15403	DRB0116	Uncharacterized conserved secreted protein	s	R	R		**63,2**	**18,7**	H	

GY15244	DRB0121	ABC-type Fe3+-siderophore transport systems, ATPase component	S	R	R	R	**26,1**	**11,8**	H	
GY14342	DRB0129	Hemolysin-like CBS domain containing protein	S	R	R	s	**49,5**	**19,7**	H	
