# Supplementary material for: Identification of New Genes Contributing to the Extreme Radioresistance of Deinococcus radiodurans Using a Tn5-Based Transposon Mutant Library
Source: PLoS One. 2015 Apr 17;10(4):e0124358. doi: 10.1371/journal.pone.0124358 (PMC4401554; doi:10.1371/journal.pone.0124358)
Supplement: S2 Table — (PDF) [file pone.0124358.s003.pdf]

Table S2. Bacterial strains and plasmids.

Strain or plasmid	Genotype or other relevant characteristics	Source or reference
**Strains**		
E. coli		
DH5α	*supE44 hsdR17 recA1 endA1 lacZ*Δ*M15*	Laboratory stock
SCS110	*endA dam dcm supE44* Δ(*lac-proAB*) (*F'traD36 proAB lacI*^{q}ZΔ*M15*)	Laboratory stock
GY11436	DH5α (*F'lacI*::*kan lacZ*$^{+}$)	Laboratory stock
XL1Blue	*endA1 gyrA96 hsdR17 lac recA1 relA1 supE44 thi-1* (*F'proAB lacI*^{q}ZΔ*M15 Tn10*)	[1]
D. radiodurans		
R1	ATCC 13939	[2]
GY10973	R1 *amyE*Ω(P_{tufA}::*lacI kan*)	[3]
GY14317	R1 Δ*pprA*Ω*hph*	This work
GY14358	GY10973 Δ*pprA*Ω*hph*	This work
GY14863	R1 Δ*DR0007*Ω*hph*	This work
GY15367	R1 Δ*DR0008*Ω*hph*	This work
GY15375	R1 Δ*DR0007*Δ*DR0008*Ω*hph*	This work
GY15378	R1 Δ*DR0009*Ω*hph*	This work
GY15313	R1 Δ*DR0265*Ω*hph*	This work
GY15451	R1 Δ*oxyR*Ω*hph*	This work
GY15452	R1 Δ*oxyR2*Ω*tetA*	This work
GY15454	R1 Δ*oxyR*Ω*hph*Δ*oxyR2*Ω*tetA*	This work
GY14960	R1 Δ*DR2462*Ω*hph*	This work
GY15316	R1 DR2462::*spa*::*cat*	This work
GY15455	R1 Δ*DR0007*Ω*hph* /p14726	This work
GY15457	R1 Δ*DR0007*Ω*hph* /p11559	This work
GY15371	R1 Δ*DR0008*Ω*hph*/p14728	This work
GY15376	R1 Δ*DR0007*Δ*DR0008*Ω*hph*/p14729	This work
GY15379	R1 Δ*DR0009*Ω*hph*/p13567	This work
GY15341	R1 Δ*DR0265*Ω*hph*/p13564	This work

GY15340	R1 Δ*DR2462*Ω*hph*/p13563	This work
**Plasmids**		
pTRC99a	Source of $lacI^q$ gene	Pharmacia
pWH1891	Source of hyperactive Tn*5* transposase	Gift of W. Hillen; [4]
p11559	Expression vector; P_{spac}-*term116*, P_{tufA}::*lacI*, Spc^R in *E. coli* and *D. radiodurans*	[5]
p11615	Source of a Tet^R cassette in *D. radiodurans*	[5]
p12625	Source of a Hyg^R cassette in *D. radiodurans*	Gift of I. Narumi
p12723	Source of *flag*-tag chloramphenicol cassette	[6]
p13841	p11830 (Spc^R RepUTs) P_{spac}-*term116*	[7]
p13537	pGY13841::$lacI^q$	This work
p13545	p13537, P_{spac}:: *tnp* (encoding hyperactive Tn*5* transposase)	This work
p13547	p13545, mini-Tn*5*-Hyg^R	This work
p13554	p13547Δ$lacI^q$	This work
p13563	p11520 with a PCR fragment encoding *DR2462*	This work
p13564	p11520 with a PCR fragment encoding *DR0265*	This work
p14726	p11559; P_{spac}::*DR0007-term116*	This work
p14728	p11559; P_{spac}::*DR0008-term116*	This work
p14729	p11559; P_{spac}::*DR0007-DR0008-term116*	This work
p13567	p11559; P_{spac}::*DR0009-term116*	This work
p14730	p11520 with a PCR fragment encoding the end of *DR0008-term116*	This work
p14731	p11520 with a PCR fragment encoding *DR0007-DR0008-term116*	This work

Supplementary references.

1. Bullock WO, Fernandez JM, Short JM (1987) XL1-Blue: a high efficiency plasmid transforming recA Escherichia coli strain with β-galactosidase selection. BioTechniques 5: 376-378.

2. Anderson AW, Nordon HC, Cain RF, Parrish G, Duggan G (1956) Studies on a radio-resistant micrococcus. I. Isolation, morphology, cultural characteristics, and resistance to gamma radiation. Food Technol 10: 575-578.
3. Lecointe F, Coste G, Sommer S, Bailone A (2004) Vectors for regulated gene expression in the radioresistant bacterium Deinococcus radiodurans. Gene 336: 25-35.
4. Köstner M, Schmidt B, Bertram R, Hillen W (2006) Generating tetracycline-inducible auxotrophy in Escherichia coli and Salmonella enterica serovar Typhimurium by using an insertion element and a hyperactive transposase. Appl Environ Microbiol 72: 4717-4725.
5. Mennecier S, Coste G, Servant P, Bailone A, Sommer S (2004) Mismatch repair ensures fidelity of replication and recombination in the radioresistant organism Deinococcus radiodurans. Mol Genet Genomics 272: 460-469.
6. Bouthier de la Tour C, Toueille M, Jolivet E, Nguyen HH, Servant P, et al. (2009) The Deinococcus radiodurans SMC protein is dispensable for cell viability yet plays a role in DNA folding. Extremophiles 13: 827-837.
7. Nguyen HH, de la Tour CB, Toueille M, Vannier F, Sommer S, et al. (2009) The essential histone-like protein HU plays a major role in Deinococcus radiodurans nucleoid compaction. Mol Microbiol 73: 240-252.
