# Supplementary material for: Identification of New Genes Contributing to the Extreme Radioresistance of Deinococcus radiodurans Using a Tn5-Based Transposon Mutant Library
Source: PLoS One. 2015 Apr 17;10(4):e0124358. doi: 10.1371/journal.pone.0124358 (PMC4401554; doi:10.1371/journal.pone.0124358)
Supplement: S3 Table — (PDF) [file pone.0124358.s004.pdf]

Table S3. Overview of primers used for strains construction, cloning, mutagenesis, and AP-PCR experiments. Oligonucleotides used to check homogenotization of backcross insertion mutants will be provided upon request.

Primer	Primers sequences (5' → 3')[a]	Use
**Construction of the mutant Δ*pprA*ΩP*kat*::*hph* (tripartite ligation procedure, strain GY14317)**		
UP347<br>347X	GGCTCCTCGATGACCGCCGCCGCCAG<br>GGTT**CTCGAG**GTAGCACTCTGGCATAAGG (c)	Amplification of the *DRA0347* portion from genomic DNA of R1
Col1918<br>HpBgl	GTCTGACGCTCAGTGGAACG<br>GCGG**AGATCT**TCCGTGTTTCAGTTAGCCTCCC (c)	Amplification of the P_{kat}::*hph* cassette from plasmid p12625
345Bgl<br>Dwn345	CTGG**AGATCT**GCGCGAGAGCTGAGTCAAG<br>GCGTTGAAGTCGCGCAGCCAGCCC (c)	Amplification of the *DRA0345* portion from genomic DNA of R1
**Construction of the mutant Δ*oxyR*ΩP*kat*::*hph* (tripartite ligation procedure, strain GY15451)**		
Up614<br>614Xho	TGCTGCCAGAGCCGCTCCTCGAAC<br>TGTAT**CTCGAG**GCCTGGGCTGTCACGAGTCGG (c)	Amplification of the *DR0614* portion from genomic DNA of R1
Col1918<br>520FOR	GTCTGACGCTCAGTGGAACG<br>AGCTATGACCATGATTACGC (c)	Amplification of the P_{kat}::*hph* cassette from plasmid p12625
615Xba<br>Dn616	TGGC**TCTAGA**AGGTGACGCGCCGCATATAAAG<br>TCAGAGAACGCTGGCGACCGCTTG (c)	Amplification of the *DR0616* portion from genomic DNA of R1
**Construction of the mutant Δ*oxyR2*ΩP*groEL*::*tetA* (tripartite ligation procedure, strain GY15452)**		
Up335<br>335Pst	GAGCTGCGGACGAACATCATC<br>TTGA**CTGCAG**GGCGACATGCAGGAAACAGGC (c)	Amplification of the *DRA0335* portion from genomic DNA of R1
UpTet<br>TetBg	AGTAGTTCGCCAGTTAATAG<br>GTGT**AGATCT**GCGGCTTCCATTCAGGTCGAG (c)	Amplification of the P_{groEL}::*tetA* cassette from plasmid p11615
337Bg<br>Dn337	GCGG**AGATCT**AGGCAGCTATTGGACATCTCGC<br>ACATAATCGCCGCCGTCCTTG (c)	Amplification of the *DRA0337* portion from genomic DNA of R1
**Construction of the mutant Δ*DR0007*ΩP*kat*::*hph* (tripartite ligation procedure, strain GY14863)**		
UpDR0007<br>DR0007Xho	CGGTAGGTGCCGCCGATGTTC<br>GGTT**CTCGAG**CAAAGACCATGATGCCGCGCAG (c)	Amplification of the *DR0006* portion from genomic DNA of R1

XhoHPH<br>HpBgl	GGC**CTCGAG**TCGCATGGAGACCGAGGG<br>GCGG**AGATCT**TCCGTGTTTCAGTTAGCCTCCC (c)	Amplification of the P_{kat}::*hph* cassette from plasmid p12625
DR0007Bg<br>DnDR0007	CTGG**AGATCT**GTCTATGAGCGCGAGGCATTG<br>CGCGTCACCTGATTGAGCAC (c)	Amplification of the *DR0008* portion from genomic DNA of R1
**Construction of the mutant Δ*DR0008*ΩP_{kat}::*hph* (tripartite ligation procedure, strain GY15367)**		
Up08<br>Dn08Xho	CTGGTGGTGCTGTTTCAGCC<br>TGTG**CTCGAG**TGCCACTGTTTGCGCTTGC (c)	Amplification of the *DR0007* portion from genomic DNA of R1
Col1918<br>520FOR	GTCTGACGCTCAGTGGAACG<br>AGCTATGACCATGATTACGC (c)	Amplification of the P_{kat}::*hph* cassette from plasmid p12625
Up009Bg<br>Dn009	TTGG**AGATCT**AGGCAGCTCATACCCGCTTC<br>CGTCCACCTTCAGGCCGTTG (c)	Amplification of the *DR0009* portion from genomic DNA of R1
**Construction of the double mutant Δ*DR0007*Δ*DR0008*ΩP_{kat}::*hph* (dipartite ligation procedure, strain GY15375)**		
UpDR0007<br>EB90	CGGTAGGTGCCGCCGATGTTC<br>AGCTCGAATTCGATATCAAG (c)	Amplification of the *DR0006* portion from genomic DNA of GY14863
Seq08FOR	GCGAAGCGCAGGGCAAGC	Amplification of a fragment including the hph cassette and the *DR0009* portion from genomic DNA of GY15367
Dn009	CGTCCACCTTCAGGCCGTTG (c)	
**Construction of the mutant Δ*DR0265*ΩP_{kat}::*hph* (tripartite ligation procedure, strain GY15313)**		
DR0265Up<br>DR0265Up Xho	GACGTGGGCAACCACAACGG<br>CATG**CTCGAG**GCTGCCTAGAGGTCATCAATCAC (c)	Amplification of the *DR0266* portion from genomic DNA of R1
XhoHPH<br>HpBgl	GGC**CTCGAG**TCGCATGGAGACCGAGGG<br>GCGG**AGATCT**TCCGTGTTTCAGTTAGCCTCCC (c)	Amplification of the P_{kat}::*hph* cassette from plasmid p12625
DR0265Dn<br>DR0265Dn Bgl	CGCGCTGCTCCTCTTCCTGC<br>CAGT**AGATCT**AGCTGCGCGGCGGCGAGTCG (c)	Amplification of the *DR0264* portion from genomic DNA of R1
**Construction of the mutant Δ*DR2462*ΩP_{kat}::*hph (*tripartite ligation procedure, strain GY14960)**		
DR2462Up Xho	CTAG**CTCGAG**GTTCTGTCTTGGCTCCTTTCGG	Amplification of the *DR2461* portion from genomic DNA of R1
DR2462Up	GCCGGCAATGGCGGCGCGCAG (c)	
XhoHPH	GGC**CTCGAG**TCGCATGGAGACCGAGGG	Amplification of the P_{kat}::*hph* cassette from plasmid p12625
HpBgl	GCGG**AGATCT**TCCGTGTTTCAGTTAGCCTCCC (c)	
DR2462Dn Bgl	CTAG**AGATCT**GCAGGCCCCTCTCCACACGC	Amplification of the *DR2463* portion from genomic DNA of R1

DR2462Dn	GGGAATGTCGCTGTCTTCAG	
**Construction of strain GY15316 (expressing the flag-tagged DR2462 protein)**		
PS212<br>PS214	GAG**GGATCC**ATGGAAAAGAGAAGATG<br>GTT**TCTAGA**CGCACTTATTCAGGCGTAGC (c)	Amplification of the spa-tag cat cassette from plasmid p12723
DR2462Dn Xba<br>DR2462Dn	GTAC**TCTAGA**AGCAGGCCCCTCTCCACACGC<br>GGGAATGTCGCTGTCTTCAG (c)	Amplification of the DR2463 portion from genomic DNA of R1
DR2462-2117<br>DR2462Bam	GGGCTGGTGCAACTGCTCGG<br>CTAG**GGATCC**GCGGGCGACCCCCACC (c)	Amplification of the 3' end of DR2462 from genomic DNA of R1
**Cloning**		
**Construction of the Tn*5* delivery vector (p13554)**		
ForLacI<br>RevLacIBg	CGCG**AGATCT**GAAGCGGCATGCATTTACGTTGAC<br>GGCC**AGATCT**TCACATTAATTGCGTTGCGCTCAC (c)	Amplification of the *lacI*^{Q} gene from plasmid pTRC99A
Tnp5UP<br>Tnp5Dra	GTTTAACTTTAAGAAGGAGATATAC<br>GGTTAC**TTTAAA**TCATATCTTGATCCCCTGCGCCATCAGATCC (c)	Amplification of the hyperactive Tn*5* transposase encoding gene from plasmid pWH1891
MEUpBst<br>MEDnBgl	GTGCCT**GTATAC**CTGTCTCTTATACACATCTATTCGAGCTCGCATGGAGACCGAGGGC<br>GTGT**AGATCT**CTGTCTCTTATACACATCTGAGGATCCGTGTTTCAGTTAGCCTCCC (c)	Amplification of the mini-Tn*5*-Hyg^{R} transposon with ME (mosaic ends) from the plasmid p12625
LinkPscA<br>LinkPscB	CATGTCGCCGGTCGACGGTGC<br>GCACCGTCGACCGGCGA (c)	Creation of a linker containing *Dra*I and *Psc*I ends and an internal *Sal*I site
**Construction of p14726 (for complementation analysis of Δ*DR0007*ΩP$_{kat}$::*hph*)**		
DR0007Nde<br>XhoDR0007	TGTC**CATATG**CCGGTGGGCGACCGAAGGAG<br>ATTA**CTCGAG**CGCCGCCGCTCATGGCCG (c)	Amplification of the *DR0007* CDS fragment from genomic DNA of R1
**Construction of p14728 (for complementation analysis of Δ*DR0008*ΩP$_{kat}$::*hph*)**		
DR0008Nde<br>DR0008Dra	TTGG**CATATG**AGCGGCGGCGAAGCGCAG<br>GCGC**TTTAAA**CTTTGCTCAATTCCCACCAC (c)	Amplification of the *DR0008* CDS fragment from genomic DNA of R1
**Construction of p14729 (for complementation analysis of Δ*DR0007*Δ*DR0008*ΩP$_{kat}$::*hph*)**		
DR0007Nde<br>DR0008Dra	TGTC**CATATG**CCGGTGGGCGACCGAAGGAG<br>GCGC**TTTAAA**CTTTGCTCAATTCCCACCAC (c)	Amplification of the fragment including both *DR0007* and *DR0008* CDS from genomic DNA of R1
**Construction of p14731 (for complementation analysis of Δ*DR0007*Δ*DR0008*ΩP$_{kat}$::*hph*)**		
Up07Sac<br>DnSacII	GCTGGAGCTCGGGTTCGATGAGCAGACGG<br>GGACCGATGTTGTGCCGCAGCC (c)	Amplification of the fragment including *DR0007* CDS and beginning of *DR0008* from genomic DNA of R1

**Construction of p13564 (for complementation analysis of Δ*DR0265*ΩP$_{kat}$::*hph*)**		
265Xba<br><br>265Bam	GTGT**TCTAGA**GCCCGTGTTGGCAGATGCCAG<br>GTGT**GGATCC**GGGCGTGAAACCAGATAAGC (c)	Amplification of the *DR0265* fragment from genomic DNA of R1
**Construction of p13563 (for complementation analysis of Δ*DR2462*ΩP$_{kat}$::*hph*)**		
2462Xba	GTGT**TCTAGA**CCCTTCTCAGACGGATTTCG	Amplification of the *DR2462* fragment from genomic DNA of R1
2462Bam	GTGTGGATCCGTTCAACATGGTAGCGCCGAG (c)	
**AP-PCR for mapping Tn*5* insertion sites**		
ARB1c<br>Tn5-212	GGCCTCGCGTCGACTACTTCN_{10}ATCGG<br>CAGGTCGGAGACGCTGTCGAAC (c)	First round of arbitrary PCR for mapping 5’ end of Tn*5*
ARB3<br>Tn5-166	GGCCTCGCGTCGACTACTTC<br>GACGTCGCGGTGAGTTCAGGC (c)	Second round of arbitrary PCR for mapping 5’ end of Tn*5*
ARB1c<br>Tn5-991	GGCCTCGCGTCGACTACTTCN_{10}ATCGG<br>ATGCAGCTTGGGCGCAGGGTC (c)	First round of arbitrary PCR for mapping 3’ end of Tn*5*
ARB3<br>Tn5-1055	GGCCTCGCGTCGACTACTTC<br>TACACAAATCGCCCGCAGAAG (c)	Second round of arbitrary PCR for mapping 3’ end of Tn*5*
**Sequencing**		
HH67	CGAGTCAGTGAGCGAGGAAG	Sequencing of *lacI*^{q} into p13537
UpNd559<br>DnTnp	AAGACCGGCCCGATCTACG<br>GCATGCCGCTTCAGATCC	Sequencing of *tnp* gene (encoding hyperactive Tn5 transposase) into p13545
SeqTn5-1<br>SeqTn5-2	GCGGTATCTACCAGCTGTC<br>CCTTGTAGATAAAGTCAAC	Sequencing of mini-Tn5-Hyg^R into p13547
SeqRE	CATATGCTCTCCTTCGCCTC	Sequencing of arbitrary PCR products (5’end of Tn*5*)
EB89	CGCCGATAGTGGAAACCGAC	Sequencing of arbitrary PCR products (3’end of Tn*5*)
Seq559Up	TTCAGAGCACGTTCCGTTTC	Sequencing of *DR0007*, *DR0008*, *DR0009* and the region *DR0007-DR0008* into p14726, p14728, p13567 and p14729 respectively
DnXho559	GACTGGAAAGCGGGCAGTG	
520FOR	AGCTATGACCATGATTACGC	Sequencing of *DR0265* and *DR2462* into p13564 and p13563 respectively
520REV	TAGCGACCAGAGTTATCTAC	

[a] tags with restriction site are in bold. (c) Sequence is on the complementary strand.
